# Navigating through toxicological data: review of toxicological databases and sources in the interdisciplinary context including—chemicals, cosmetics, pharmaceuticals and forensics

**DOI:** 10.1007/s00204-026-04379-y

**Published:** 2026-04-20

**Authors:** Kamil Jurowski, Łukasz Niżnik, Maciej Noga, Damian Kobylarz, Adrian Frydrych, Oktawia Fijałkowska, Eugenie Nepovimova, Kamil Kuca

**Affiliations:** 1 Department of Toxicology, Faculty of Medicine, Collegium Medicum, Rzeszów University, Al. mjr. W. Kopisto 2a, 35-959 Rzeszów, Poland; 2 Department of Regulatory and Forensic Toxicology, Institute of Medical Expertises in Łódź, ul. Aleksandrowska 67/93, 91-205 Łódź, Poland; 3 Laboratory of Toxicological Research and Analyzes, Center for Innovative Research in Medical and Natural Sciences, Faculty of Medicine, Medical College, Rzeszów University, Al. mjr. W. Kopisto 2a, 35-959 Rzeszów, Poland; 4 Centre for Basic and Applied Research, Faculty of Informatics and Management, University of Hradec Kralove, CZ-500 03 Hradec Kralove, Czech Republic; 5 Biomedical Research Center, University Hospital Hradec Kralove, Hradec Kralove, Czech Republic; 6 Center of Advanced Innovation Technologies, VSB-Technical University of Ostrava, 708 00 Ostrava-Poruba, Czech Republic; 7 Department of Chemistry, Faculty of Science, University of Hradec Kralove, 50003 Hradec Kralove, Czech Republic

**Keywords:** Toxicological databases, Regulatory toxicology, Safety assessment, Toxicological data

## Abstract

Toxicology, an interdisciplinary and critical field in environmental and public health safety, is based heavily on the comprehensive analysis and application of toxicological data. This review addresses the use and visibility of toxicological databases that are instrumental in the assessment of chemical risks and the information of regulatory decisions. Despite the critical nature of these databases, there is a notable gap in their effective use within the regulatory and scientific communities. Our systematic overview of existing toxicological databases highlights the significant resources available, including databases that consolidate vast amounts of chemical hazard, exposure, and risk assessment data. We categorise these databases based on their functionalities, discuss the challenges associated with their use, and propose solutions to enhance their accessibility and integration across different platforms. This review not only underscores the importance of these databases in shaping informed regulatory policies, but also sheds light on the need for improved standardisation and technological enhancements to maximise their potential. The findings aim to foster a deeper understanding among toxicologists, regulators, and policymakers, ultimately contributing to stronger and more effective public health protections.

**Supplementary Information:**

The online version contains supplementary material available at 10.1007/s00204-026-04379-y.

## Introduction

Toxicology, the branch of science concerned with the nature, effects, and detection of poisons, is a cornerstone of public and environmental health safety. Its applications in regulatory toxicology are crucial for setting and enforcing standards that mitigate risks associated with chemical exposures. Despite the critical importance of these applications, regulatory toxicologists and stakeholders often navigate a challenging landscape characterised by an abundance of chemical substances and associated data. To effectively manage these challenges, a comprehensive and accessible array of toxicological databases is essential (Petit 2022; Yang et al. 2006). 

There is no doubt that toxicological databases play an essential role in the field of regulatory toxicology (Benigni et al. 2013), providing critical information that supports the safety assessment and regulatory approval of chemicals and pharmaceuticals. The availability of comprehensive, accurate, and accessible toxicological data is essential to understand the potential health risks associated with exposure to various substances (Russom 2002). These databases facilitate efficient evaluation of toxicological profiles, help design safer chemicals, and comply with regulatory standards (Judson 2010).

The importance of toxicological databases in the regulatory context cannot be overstated. They are foundational tools that allow risk assessors and regulatory bodies to make informed decisions, ensure public health safety, and promote environmental protection. Moreover, these databases support the advancement of nonanimal testing methodologies by providing data that can be used in computational models and in vitro tests, aligning with the ethical push towards reducing animal testing in toxicological research.

Surprisingly, no comprehensive review article has been published that collates and evaluates the currently active toxicological databases. The absence of such a review has been a notable gap in the literature, as researchers, regulators, and industry stakeholders must have a clear overview of available resources, their scope, and their utility in various regulatory frameworks.

This pioneering article addresses this gap by thoroughly analysing existing toxicological databases. This article is not only timely, but also essential, marking a significant contribution to the field of toxicology. Enhances the accessibility of information, guiding users on how to navigate and use these resources for regulatory purposes effectively. The exploration of the databases in the article, coupled with critical insights into their application in a regulatory setting, adds tremendous value to the discipline, helping to globalise safety assessment practices. 

This review article is important in clarifying the landscape of toxicological databases, underscoring their importance and utility in regulatory toxicology. It serves as a crucial resource for all stakeholders involved in the safety assessment of different topics (chemicals, cosmetics products, pharmaceuticals/drugs, forensics, food, environmental toxicology, and ecotoxicology), offering guidance and fostering a deeper understanding of how to leverage these tools for improved health outcomes and regulatory compliance.

## Materials and methods

### Selection of toxicological databases

In conducting this review, we meticulously selected key toxicological databases in four distinct but interrelated areas of toxicology: regulatory, forensic, food, and environmental and ecotoxicology. This selection process aimed to cover the most significant and largest databases relevant to each speciality, ensuring a comprehensive exploration of the resources available to professionals, including regulatory toxicology, forensic toxicology, food toxicology, environmental toxicology, and ecotoxicology.

Our regulatory toxicology focus was on databases that provide robust information for the development, evaluation and implementation of chemical regulations and standards. These databases are essential for toxicologists and regulatory bodies to ensure public safety against hazardous substances. Selected databases offer access to a wide range of data on chemical hazards, risk assessments, exposure levels, and compliance with international regulatory frameworks.

The databases in forensic toxicology support the analysis of chemicals, drugs, and toxins in legal and criminal investigations. These databases provide crucial information on drug interactions, pharmacokinetics, and physiological effects of toxins and substances.

For food toxicology, the selected databases specialise in information on contaminants, food additives, pesticide residues, and veterinary drug residues in food products. These resources are vital to ensure food safety and to assess potential health risks from food consumption.

In environmental toxicology and ecotoxicology, we prioritise databases that offer insights into the impacts of chemicals on ecosystems and wildlife. These databases provide essential data on the persistence, bioaccumulation, toxicity, and ecological consequences of chemical exposures.

This review aims to illuminate the essential tools available to professionals in various branches of toxicology by selecting these databases. Each database has been chosen based on its relevance, breadth of data, and specificity to the needs of the toxicological community, ensuring that the resources highlighted are comprehensive and of the highest utility in their respective fields.

### Evaluation of database content

In our systematic evaluation of toxicological databases, we used a structured methodology based on predefined criteria to assess their relevance, completeness, precision, and utility. Relevance was measured by aligning the content of the database with current regulatory needs and research questions in toxicology. At the same time, comprehensiveness was judged based on the scope of information provided, including chemical hazards, exposure levels, risk assessments, and regulatory status. The accuracy of the databases was evaluated by examining the sources, validation methods and the frequency of update of the data. The utility was evaluated through ease of access to the databases, user interface design, search functionality, and available support resources. Our findings indicate that while many databases effectively meet contemporary needs for toxicological research and regulation, gaps remain, particularly in the coverage of emerging chemicals and user interface accessibility. Some databases demonstrated robust, user-friendly interfaces and provided comprehensive, accurate, and regularly updated content, while others were hampered by outdated information and less intuitive navigation. To enhance the efficacy and reach of these toxicological databases, we recommend developing interoperability standards for data integration, redesigning user interfaces to improve accessibility, and establishing regular review protocols to ensure the accuracy and relevance of the data. These improvements are critical to enable more informed regulatory decision-making and advance the field of toxicology.

### Presentation of the results

In the manuscript detailing the evaluation of toxicological databases, we present the results in a structured manner that provides a clear and concise overview of each database, focussing on their relevance, comprehensiveness, accuracy, and utility. The relevance of each database is evaluated to determine how well it aligns with current toxicological research needs and regulatory requirements, highlighting its applicability to modern safety assessments. The comprehensiveness of the databases was evaluated by examining the range of toxicological information provided, including the spectrum of chemicals covered and the types of data available, such as risk assessments and toxicological endpoints.

## Toxicological databases important for regulatory toxicology

### Databases useful for chemical assessment

Electronic methods are essential for managing the diverse information on chemical compounds or drugs, including the literature, physicochemical properties, and reactions (Engel and Zass 2007). These methods, often called information systems, consist of application programmes such as search engines, database management systems, and organised data collections known as databases. In chemistry, the term “database” is sometimes incorrectly used to refer to the entire information system, a database system, or a data file. Information systems utilise two main approaches to provide data from a database. One approach involves using a database system (DBS) that efficiently organises, generates, manipulates, and manages a large collection of data. The database is a storage component within the system, known as a “container.” Another crucial software component is the DataBase Management System (DBMS) such as Oracle, DBase, or MS Access. This software enables data storage based on a structured database, facilitating data retrieval, manipulation, user management, security, backup, and load balancing. As the methods to calculate the heat of formation of molecules advanced, the importance of large sets of experimental data for small molecules grew significantly (Morgante and Peverati 2019). Early semiempirical methods paved the way for more sophisticated Gaussian (Curtiss et al. 2011) composite methods and multicoefficient correlation methods (Fast et al. 2001). However, recent developments in high accuracy ab initio calculation methods have shifted focus towards databases of purely calculated data. These databases offer clear advantages for assessing and parameterising semiempirical electronic structure methods. They provide directly comparable data between calculations, eliminating the need for corrections to experimental conditions and reducing errors associated with experimental measurements. Although they still face computational accuracy errors, these are easier to assess and control than stochastic experimental errors, making high-level calculated data preferable in many cases. 

#### ECHA

The European Chemical Agency (ECHA), headquartered in Helsinki, Finland, offers a suite of tools and services such as CHEMSAR portal, ECHA CHEM, ECHA Cloud Services, ePIC, EU Observatory for Nanomaterials, EUSES (European Union System for the Evaluation of Substances), Interact Portal, IUCLID 6, Poison Control Centres, QSAR Toolbox software applications, R4BP 3, REACH-IT, S2S, SCIP and SPC (ECHA 2024). To ensure accessibility, ECHA provides online content and public documents in all official languages of the EU. Regular updates on substances added to their database and relevant news are available on the ECHA website, aligned with the REACH and CLP regulations (ECHA 2024). ECHA contributes to the Sustainable Development Goals of the United Nations by providing comprehensive chemical information, including classification, labelling, hazardous properties, and safe use. Additionally, ECHA collaborates with Member States Competent Authorities to prepare Annex XV dossiers identifying Substances of Very High Concern (SVHCs) upon request by the Commission. These include CMR substances, PBT/vPvB substances, and those identified as endocrine disruptors based on scientific evidence. ECHA also handles Annex XV transitional reports for existing substance risk assessments and risk reduction strategies under Regulation (EEC) No. 793/93, which was not finalised by June 1, 2008. Regarding specific collections, the ECHA Dossier Evaluation Status involves compliance checks and examination of testing proposals, with decisions published after consultation and redaction of personal data (Enhesa 2024). The RMOA Hazard Assessment Activities focus on completed risk management options analyses for PBT/vPvB or endocrine disruptor properties since February 2013. Lastly, the SVHCs in the Consumer Articles collection contain submissions from companies notifying ECHA of Candidate List substances present in their consumer articles, as mandated by REACH regulations.

#### ToxPlanet

ToxPlanet offers convenient access via mobile devices, although significant results set and documents may not be ideal for mobile viewing. The sharing of subscriptions is prohibited and users must adhere to the contracted number of users (Enhesa 2024). Logging in is straightforward through the website’s Customer Login option using the provided credentials.

ToxPlanet offers authoritative and comprehensive content from hundreds of reputable sources worldwide, covering various subject areas, including chemical hazards and toxicology. It boasts more than 9.4 million documents, including analytical methods, hazard assessments, regulatory submissions, and more. The content is regularly updated within 72 h of new information becoming available, and customer requests for new content are welcomed. ToxPlanet’s international scope includes data from leading global organisations such as ATSDR, ECHA, and WHO. Users can access additional support documents from industry-leading sources through the Help menu. The content is sourced from private providers and government agencies, ensuring a broad and reliable information base that meets the needs of professionals worldwide.

The ToxPlanet content is authoritative, comprehensive, and regularly updated, covering various chemical information needs. Users can access support documents and provide feedback or suggestions to improve the platform. Its benefits include authoritative content, currency, relevance, efficiency, searchability, speed, convenience, discovery, and comprehensiveness, catering to chemical safety, research professionals, and other users seeking reliable chemical hazard and toxicology information.

ToxPlanet offers a diverse range of products tailored to the needs of chemical safety professionals. ChemEXPERT™ provides access to integrated content from over 175 leading chemical hazard sources, while DrugEXPERT™ offers detailed environmental health and safety data on pharmaceutical substances. ListEXPERT™ contains data on chemicals from regulatory lists worldwide, and MSDSonline^®^ is a vast collection of safety data sheets. PoisonEXPERT™ offers poison management recommendations, and REACH Registrations provides access to the official ECHA REACH Registrations Database. ReproEX-PERT™ delivers human reproductive risk data, while Similar Compounds automates the search for structurally-similar compounds. The EXPERTIndex™ facilitates efficient chemical searching, and the TOX-LINE^®^ Special covers the biochemical, pharmacological, and toxicological effects of drugs and chemicals. The Environmental Chemistry Information System (ECIS) offers substance-specific collections for environmental health and safety professionals. If particular product tabs appear greyed out, it may indicate that those products do not contain relevant content for the conducted searches or are not included in the subscription.

ToxPlanet offers personalised search through MyEXPERT™, enabling users to customise their search results by defining preferred collections. Data gaps are addressed using the similar compounds feature, which automates the search for structurally similar compounds. Search results are ensured to be relevant through the EXPERTIndex™, providing consistent and finetuned results. To facilitate discovery, ToxPlanet allows simultaneous searches across all content collections, revealing valuable expected and previously unknown information. Seven search modes cater to various search preferences, including EX-PERTIndex™ Search, Full Text Search, MSDSonline^®^ Advanced, TOXLINE^®^ Special Advanced, REACH Registrations Advanced, TSCATS Complete™ Advanced, and ListEXPERT-List View.

ToxPlanet provides users with comprehensive tools to manage their usage and access content effectively. Users can generate usage reports to track their activity, review recently opened documents, and perform searches. Instructional support is readily available in various formats to help users navigate the platform efficiently. Additionally, guidelines are provided for referencing ToxPlanet content in documents or publications, ensuring proper citation.

#### Spectral database for organic compounds (SDBS)

SDBS (Spectral Database for Organic Compounds) is an extensive integrated database system for organic compounds, offering six types of spectra for each compound (AIST 2024). Developed in the early 1970s, it underwent construction and maintenance by the National Metrology Institute of Japan (NMIJ) starting in 2001. Currently, the database includes Electron Impact Mass Spectrum (EI-MS), 1 H Nuclear Magnetic Resonance (NMR), 13 C NMR, Fourier Transform Infrared Spectrum (FT-IR), and Raman spectra. With approximately 34,600 compounds and more than 55,000 spectra, SDBS provides vital information for researchers around the world. Spectra are measured under specific conditions for each type, ensuring precision and reliability. Users can access the database for free through the Tsukuba Advanced Computing Center (TACC). Although the database mainly comprises commercial chemical reagents, efforts have been made to maintain high-quality data.

#### CAMEO

CAMEO Chemicals Software, a part of the CAMEO^®^ software suite, is a comprehensive hazardous chemical database for planning and responding to chemical emergencies (US Epa 2017). It includes a feature to predict potential hazards resulting from chemical mixtures. Users can access chemical datasheets that contain critical response details such as physical properties, health hazards, air and water hazards, and recommendations for firefighting, first aid, and spill response. Additionally, datasheets based on UN/NA identification numbers offer response information from the Emergency Response Guidebook (ERG) and shipping details from the Hazardous Materials Table (49 CFR 172.101). CAMEO Chemicals is accessible through a website, a mobile site, a mobile app, and a desktop programme. The mobile app and the desktop version can run offline, and the desktop programme can share information with other CAMEO suite programmes.

#### ISSTOX

ISSTOX is a group of toxicological databases developed by the Istituto Superiore di Sanità. Features freely accessible databases that use modern information technologies and meticulous curation of biological data quality. The ISSTOX databases are designed for various toxicological endpoints and are integral to research and regulatory evaluations involving chemical mutagenicity and carcinogenicity. The structure of these databases allows for efficient data mining and supports multiple research and regulatory functions, such as QSAR studies for the prediction of toxicity and the development of new toxicity testing strategies.

The databases included in ISSTOX are:

ISSCAN: Focusses on long-term carcinogenicity bioassay results in rodents (rats and mice).

ISSSTY: Contains in vitro mutagenesis results from the Ames test using Salmonella typhimurium.

ISSMIC: Details of in vivo mutagenesis assays, specifically the micronucleus test.

ISSCTA: Includes in vitro cell transformation assays.

ISSBIOC: Covers the mutagenicity and carcinogenicity results for biocides and plant protection products.

These databases are organised to facilitate searches using chemical structures as identifiers, which enhances the linkage of chemical toxicity data with chemical structure information. This organisation supports advanced operations such as retrieving specific chemicals, describing database contents, finding similar chemicals, and integrating biological and chemical data for comprehensive assessments.

#### Toxin and toxin target database (T3DB)

The Toxin and Toxin Target Database (T3DB), soon known as the Toxic Exposome Database, is a distinctive bioinformatics tool that integrates comprehensive toxin data with detailed target information. The database comprises 3678 toxins documented under 41,602 synonyms, encompassing pollutants, pesticides, pharmaceuticals, and food toxins linked to 2073 corresponding toxin target records. In total, there are 42,374 associations between toxins and their targets. Each toxin record (ToxCard) encompasses more than 90 data fields, housing details such as chemical properties, toxicity metrics, molecular and cellular interactions, and medical information. These data originate from over 18,143 sources, including various databases, government documents, literature, and books (Wishart et al. 2015). The primary focus of T3DB lies in elucidating the mechanisms of toxicity and identifying target proteins for each toxin. Its distinctive feature lies in the interactive linkage of toxin and toxin target records in both directions, which sets it apart from other databases. T3DB is fully searchable and supports various queries, including text, sequence, chemical structure, and relational searches. It is designed in alignment with the Human Metabolome Database (HMDB) and DrugBank, facilitating seamless integration and comparison. Potential applications of T3DB include prediction of toxin metabolism, prognosis of toxin-drug interaction, and increasing public awareness of toxin hazards, making it beneficial in multiple domains. In general, the diversity and accessibility of T3DB make it an invaluable asset for novice users and seasoned researchers (Lim et al. 2010).

#### Tox21

Tox21 is a collaborative initiative among multiple federal agencies to develop new methods to assess whether substances adversely affect human health rapidly. Established in 2008, Tox21 involves agencies such as the NIH, including the NTP and the National Human Genome Research Institute’s National Chemical Genomics Center (NCGC), along with the EPA’s National Center for Computational Toxicology (now part of CCTE). The FDA joined the collaboration in 2010, and in 2015, the Tox21 Consortium reaffirmed its commitment by signing a new memorandum of understanding (MOU) (Thomas 2018). The primary objective of Tox21 is to research, develop, evaluate, and implement innovative test methods to improve predictions of how substances can affect humans and the environment. This includes the use of robotics for high-throughput screening (HTS) and the use of other novel methodologies to assess the toxicity of substances. Using robotics in HTS is crucial for increasing testing speed and volume, offering an alternative to conventional preclinical tests that often use animals. These conventional methods can be slow and costly and may not reliably predict toxic health effects in humans observed in clinical trials (Richard et al. 2021). Tox21 seeks to prioritise substances for further toxicological evaluation, identify mechanisms of action, develop predictive models to assess biological responses, use testing methods involving human cells (in vitro approaches), and reduce the time, effort and costs associated with testing. Additionally, the initiative aims to contribute to the reduction, refinement and replacement of animals used in toxic tests.

#### International uniform chemical information database (IUCLID)

IUCLID, the International Uniform Chemical Information Database, is a software designed to gather, store, manage, and share data concerning chemicals’ intrinsic and hazardous properties. IUCLID serves as a tool for data submission, aligning with regulatory frameworks like the European REACH Regulation and various chemical evaluation programs such as the HPV Chemicals Program, US HPV Challenge Program, and EU Biocides. Harmonised templates established within IUCLID by the OECD ensure a consistent reporting approach across different regulatory initiatives. The XML schema used in these templates facilitates electronic data exchange between IT systems. The management of the IUCLID project is a collaborative effort between the OECD and ECHA, with the support of the entire IUCLID user community (Sobanska and Le Goff 2014). The IUCLID (International Uniform Chemical Information Database) offers customisation options to manage chemical data in various contexts, providing a platform that employs globally standardised data elements relevant to chemicals. With the release of IUCLID version 6 in 2016, greater customisation capabilities were introduced, allowing extension and integration with other tools. For instance, standard IUCLID data elements can be expanded to capture specific information tailored to legislative data requirements before being incorporated into Official Harmonised Templates (OHTs). This document outlines four key aspects of IUCLID customisation: configuration and customisation of the IUCLID format, main features and addons, integration with other systems, and development of alternative user interfaces. It also highlights that customising these elements requires different expertise and resources and incorporates user stories on IUCLID customisation and data migration from ECHA, Australia, and Canada. Following a software update for IUCLID 6, a second edition of the report was published in June 2021 to reflect the latest features and processes (OECD 2019).

#### CompTox chemicals dashboard

The CompTox Chemicals Dashboard forms part of a suite of databases and web applications developed by the U.S. Environmental Protection Agency (EPA) to support its research efforts in developing and applying new approach methods (NAMs) (US Epa 2023). As the primary web-based application, the CompTox Chemicals Dashboard offers access to data and algorithms from the EPA Center for Computational Toxicology and Exposure. Since its inception in April 2017, it has undergone ten releases, progressively expanding the available data from an initial 560k data points to a total of 906k as of the December 2021 release. The dashboard encompasses over 310 individual chemical lists, encompassing various substances, including pesticides, disinfectant byproducts, and PFAS. The latest release signifies a significant architecture and design overhaul, incorporating datamart datahub technology for data management, a robust API, and a modern user interface design. This architecture facilitates the development and maintenance of new applications utilising the same foundational data and API while delivering notable performance enhancements. For instance, batch searches and downloads for 5000 chemicals are typically completed in less than 5 s, a significant improvement compared to over a minute in the previous version. Furthermore, Generalized Read-Across (GenRA) and Abstract Sifter are now distinct modules, expanding their utility for querying chemicals beyond those available in the underlying database (Williams et al. 2017).

Under various federal regulations, the EPA is tasked with many responsibilities to protect public health and the environment from the unintended repercussions of chemical use. Other government bodies also make similar decisions regarding chemicals at the federal, state, and international levels, as well as by industry stakeholders. To aid in these efforts, EPA researchers are consolidating available chemical data, including physicochemical properties, environmental behaviour, exposure levels, usage patterns, toxicity profiles, and bioassay results, into an online resource known as the Computational Toxicology (CompTox) Chemicals Dashboard, previously referred to as the Chemistry Dashboard. This tool is designed to help decision makers and scientists in quickly and effectively assessing numerous chemicals. The dashboard encompasses a vast repository of information on chemistry, toxicity, and exposure for over one million chemicals, organised into more than 300 chemical lists based on structural similarities or categorical classifications. The data and predictive models housed within the dashboard help identify chemicals that warrant further scrutiny and contribute to efforts to minimise reliance on animal testing for chemical evaluation. Users can navigate the Dashboard by chemical identifiers (e.g., DTXSID and CASRN), consumer product categories (allowing for exploring chemicals commonly found in specific types of products), and assays/genes linked to high-throughput screening data. High-throughput screening involves subjecting living cells or proteins to various chemicals and observing results indicative of potential biological responses. Furthermore, the advanced search functionality enables users to query chemicals based on their mass or molecular formula. On the contrary, batch searches can be conducted using chemical identifiers, MS-ready formulas, exact formulas, and monoisotopic mass (Sinclair et al. 2022).

The CompTox Chemicals Dashboard is a public repository of chemical data that offers a comprehensive resource for chemistry, toxicity, and exposure information for over a million chemicals. The available data encompasses diverse aspects such as chemical properties, environmental fate and transport, hazard indicators (e.g., point of departure, legacy toxicity values, screening levels, exposure limits), in vitro to in vivo extrapolation (IVIVE), exposure predictions (e.g., associated with the ExpoCast effort, use categories, toxic release inventory, monitoring data), and bioactivity data (e.g., high-throughput data from ToxCast and Tox21 efforts). Additionally, the Dashboard details similar chemicals and related substances, chemical lists, and links to reputable external resources, including open source widgets and tools such as PubChem for Bioactivities, Articles, and Patents.

#### Acute toxicity database

The subsequent database compiles the results of the aquatic acute toxicity assessments performed by the USGS CERC in Columbia, Missouri (USGS 2024). These tests serve as an initial reference point for evaluating contaminant hazards and are mandated for federal chemical registration programmes like the Federal Insecticide Fungicide Rodenticide Act (PL 80–104), amended by the Federal Environmental Pesticide Control Act of 1972 (7 U.S.C. 136-136y), and the Toxic Substances Control Act of 1976 (PL 94–469). Originally established in 1986 by Foster L. Mayer (U.S. Fish and Wildlife Service) and Mark R. Ellersieck (University of Missouri, Columbia, MO), the database encompasses 4901 acute toxicity tests conducted by CERC since 1965, involving 410 chemicals and 66 species of aquatic organisms. A publication by Mayer and Ellersieck (1986) offers an analysis of the initial 4901 toxicity experiments, using various statistical methodologies for taxonomic comparisons and assessing the impact of various factors (e.g., static versus flow-through conditions, age of test solutions, pH, temperature, water hardness and diet) on toxicity levels (Mayer and Ellersieck 1986).

#### REACH

The REACH database, established under the European Chemicals Agency (ECHA), is crucial in chemical safety and regulation. This database encompasses approximately 800,000 studies on 10,000 chemicals, making it the largest toxicological database in the world (Hartung 2016). Its comprehensive scope aims to facilitate a better understanding of the chemical universe, especially concerning animal test data submitted to the public database of ECHA. A significant aspect of the utility of the REACH database is its role in promoting alternative methods to animal testing. The database enables ‘read-across,’ a method where data from similar chemicals are used to predict the properties of untested chemicals, thereby reducing the need for new animal tests. This approach saves time and resources and aligns with ethical considerations against animal testing. The database’s design and application are supported by advanced tools like REACH-across™, which assist in navigating and interpreting the vast data. These tools are critical for stakeholders, particularly in complying with regulatory requirements and improving the accuracy of chemical assessments. Overall, the REACH database represents a paradigm shift in chemical safety assessment. Big data and computational methods are increasingly replacing traditional experimental approaches, offering a more sustainable and ethical route forward in chemical testing.

#### ChemIDplus

ChemIDplus, maintained by the National Library of Medicine, is a critical resource that provides extensive chemical information. This database is designed to support the identification and understanding of chemical substances used in environmental health and toxicology. ChemIDplus is not just a repository of chemical names and structures; it integrates multiple data types, including molecular formulas, properties, and safety information, all of which are crucial for research and regulatory requirements. The key features of ChemIDplus include its link to other NLM databases, such as TOXNET, facilitating the exploration of comprehensive health-related chemical data. This integration helps researchers and professionals in toxicology and environmental health access relevant information efficiently, such as toxicological profiles, chemical safety information, and regulatory statuses. The advanced search capabilities of the database allow users to search by structure, name, molecular weight, CAS registry numbers, and other chemical identifiers, making it a vital tool for chemical and pharmaceutical research (MSLIS 2006). Moreover, ChemIDplus serves an educational purpose by providing access to many chemical structures and their associated information, which can be used in academic settings to teach chemistry and pharmacology. The database is frequently updated to ensure that the chemical information remains current and useful for ongoing research and education. Researchers rely on this database to cross-reference safety data sheets and understand complex chemical properties essential for laboratory experiments and new drug developments. With regard to its broader impact on scientific research, ChemIDplus has been mentioned in numerous studies as a reliable chemical data source. It is essential for the development of new drugs and the conduct of risk assessments in environmental health sciences. It is particularly noted for its comprehensive data on chemical properties and interactions, which are crucial for modelling and simulations in chemical informatics (Wexler 2004).

#### Toxicity reference database (ToxRefDB)

This repository includes materials to support the release of Toxicity Reference Database (ToxRefDB) v2.1 alongside archival data (Watford et al. 2019). ToxRefDB comprises data from more than 5900 in vivo studies conducted according to guidelines or similar standards. This database provides scientists and the general public with access to a vast collection of meticulously curated animal toxicity test results, serving as a valuable resource for various retrospective and predictive toxicology endeavours. ToxRefDB follows guidelines established by the US Environmental Protection Agency and the National Toxicology Program and creates a publicly accessible platform for training and validating predictive models. The development of ToxRefDB version 2.0 (ToxRefDBv2) is detailed herein. Endpoints were categorised (e.g., required, optional) based on guidelines for various study designs, including subacute, subchronic, chronic, developmental, and multigenerational reproductive tests, distinguishing between adverse outcomes and untested conditions. Quantitative data were extracted, and dose-response modelling for nearly 28,000 datasets covering approximately 400 endpoints was conducted using Benchmark Dose (BMD) Modeling Software, with results stored for reference. The implementation of a controlled vocabulary contributed to improved data quality. At the same time, standardisation to guideline specifications and integration with the United Medical Language System (UMLS) facilitated connections between ToxRefDBv2 observations and vocabularies linked to UMLS, such as PubMed medical subject headings. As a result, ToxRefDBv2 offers increased interoperability with other resources and significantly improves the quantitative and qualitative utility of predictive toxicology research.

#### Chemical effects in biological systems database (CEBS)

The Chemical Effects in Biological Systems database (CEBS) stands as a comprehensive and distinctive resource in the field of toxicology, gathering individual and summarised animal data from the National Toxicology Program (NTP) testing initiative and various other contributors into a unified electronic repository (Lea et al. 2017). CEBS has recently undergone notable enhancements and currently encompasses more than 11,000 test articles (exposure agents) and more than 8,000 studies, incorporating all available NTP studies on carcinogenicity, short-term toxicity, and genetic toxicity. Data submitted to CEBS undergo rigorous manual curation, accession, and quality assurance reviews prior to their release to ensure superior quality. The CEBS database comprises two primary components: data collection and data delivery. To accommodate the diverse range of data generated by NTP, the data collection segment of CEBS employs an integrated relational design, offering the flexibility to capture various electronic data formats. On the other hand, the data delivery segment consists of a set of dedicated user interface tables containing preprocessed data that support each aspect of the user interface. Notably, the user interface has been updated to incorporate nine Guided Search tools, facilitating access to NTP summary and conclusion data and extensive non-NTP datasets. The CEBS system also includes the Biomedical Investigation Database (BID), a relational database responsible for loading, curating, and exporting study data to CEBS. BID is adept at capturing and displaying diverse data types, such as PCR data and additional fields of interest, including those defined by the HESI Toxicogenomics Committee. The BID has been shared with both Health Canada and the US Environmental Protection Agency (Waters et al. 2008). The CEBS database is available online at https://cebs.niehs.nih.gov/cebs/.

#### SciFinder

SciFinder, managed by the Chemical Abstracts Service (CAS), is a comprehensive database encompassing various chemical and bibliographic information relevant to numerous scientific and biomedical fields, focussing on chemistry. Originally established to aid in literature searches and provide detailed information on chemicals, drugs, and various substances, SciFinder has become a crucial resource for researchers and students alike. It offers access to two main databases: CAPLUS, which includes a wide range of journal articles, patents, conference proceedings, and more, and MEDLINE, which specialises in biomedical literature. The system has been particularly noted for its refined search capabilities and detailed bibliometric analysis, making it a preferred choice in academic and research settings (Baykoucheva 2011; Gabrielson 2018). Moreover, SciFinder supports educational endeavours through resources such as on-line tutorials tailored for specific classes such as organic chemistry. These tutorials, developed collaboratively by librarians and faculty, leverage software such as Adobe Captivate to create accessible, user-friendly guides that allow students to navigate and extract necessary information independently (Dawson et al. 2010).

#### Australian national industrial chemicals notification and assessment scheme (NICNAS)

The Chemical Assessment Reports Database of the Australian National Industrial Chemicals Notification and Assessment Scheme (NICNAS) is a significant resource established by the Australian Government to monitor and regulate chemical safety (AICIS, 2022). It contains thousands of detailed reports on industrial chemicals, detailing their properties, uses, and potential health and environmental impacts. These reports are vital for regulatory bodies, chemical manufacturers, industrial users, and researchers, helping to comply with safety standards and supporting public health and environmental initiatives (Gabriela et al. 2022). One of the key advantages of the NICNAS database is its comprehensive coverage of chemicals, which provides extensive data on safety profiles. This helps stakeholders meet Australian regulatory requirements, ensuring the safety of chemical products in the market. Assessments are based on rigorous scientific evidence, adding credibility and reliability to the information. However, the database also has limitations. The complexity of the data can make it difficult for those without specialised training to interpret, potentially limiting its utility. Updates to the database, while regular, can sometimes be delayed, which can affect stakeholders who rely on the most current information. This database has recently updated its website content with regard to categorisation, reporting, and record-keeping obligations, reflecting changes announced to take effect on April 24, 2024. These amendments align with the latest updates to the Industrial Chemicals (General) Rules 2019 and the Industrial Chemicals Categorisation Guidelines. The updates are designed to simplify compliance for chemical introducers and improve the protection of public health and the environment from hazardous chemicals. Additionally, the database’s focus on Australian regulations may not be as applicable in international contexts where regulatory frameworks differ.

#### International programme on chemical safety (IPCS)

IPCS INCHEM is a database developed through the collaborative efforts of the International Programme on Chemical Safety (IPCS) and the Canadian Centre for Occupational Health and Safety (CCOHS) (CCOHS 2024). This initiative responds to a priority action identified by the Intergovernmental Forum on Chemical Safety (IFCS) to consolidate and provide public access to internationally peerreviewed chemical safety related publications and database records from international bodies. vIPCS INCHEM offers rapid electronic access to thousands of searchable full text documents focussing on chemical risks and sound chemical management. This resource helps countries in fulfilling their commitments under UNCED’s Agenda 21, Chap. 19, which is concerned with managing toxic chemicals and hazardous wastes. It includes a range of documents such as Concise International Chemical Assessment Documents (CICADS), Environmental Health Criteria (EHC) monographs, Health and Safety Guides (HSGs), and International Chemical Safety Cards (ICSCs), among others. The CICADS, for example, provides concise yet comprehensive evaluations of the potential effects of chemicals on human health and the environment. They are developed based on selected national or regional evaluation documents or existing EHCs and undergo extensive peer review by internationally selected experts before publication. The primary objective of these documents is to characterise the hazards and dose response from exposure to chemicals, including critical studies in sufficient detail to support the conclusions drawn. Environmental Health Criteria (EHC) monographs are another cornerstone of IPCS INCHEM. Issued by the World Health Organization, these monographs provide a scientific evaluation of the risk of a wide range of chemicals, informing the establishment of safety standards and regulations. They include detailed reviews of physical and chemical properties, sources of exposure, and potential health effects. Additionally, this database includes outputs from the Harmonization Project, which aims to harmonise global approaches to chemical risk assessment. This project improves understanding of basic risk assessment principles and develops international guidance documents on specific issues, promoting transparency, and reducing unnecessary chemical testing.

#### U.S. national toxicology program (NTP)

The U.S. National Toxicology Program (NTP) is a federal program headquartered at the National Institute of Environmental Health Sciences (NIEHS), which itself is a part of the National Institutes of Health (NIH) (Bucher and Birnbaum 2016). The NTP is responsible for conducting and coordinating research on the potential health effects of exposure to various substances found in the environment, including chemicals, drugs, and other agents. The NTP conducts various studies, including laboratory experiments on animals, epidemiological research in human populations, and other investigations to evaluate the toxicity or other health effects of different substances (both cancer and noncancer). Their findings are published in the “NTP Technical Reports” reports. They are widely used by scientists, policymakers, and the public to understand the potential risks associated with exposure to various chemicals and other agents. These reports are available online: https://ntp.niehs.nih.gov/publications/reports/tr?type=Technical%20Report. NTP is advancing toxicology by incorporating functional omic technologies such as transcriptomics. Their genomic dose-response modelling approach focusses on determining the biological potency of the substances tested. NTP has developed BMDExpress 2.0 software to implement this approach and aid in data visualisation. This method is expected to improve consistency in reporting genomic dose-response data and facilitate its use in risk assessment (National Toxicology Program (NTP) 2018).

#### Comparative toxicogenomics database (CTD)

The Comparative Toxicogenomics Database (CTD) is a comprehensive platform that consolidates various data on chemical exposures and their biological effects by meticulously collecting and connecting information from the scientific literature. The meticulously curated data is used to gain insights, pinpoint potential molecular influencers, and bridge knowledge gaps in relation to environmental health. The CTD is a reservoir of information and a hub for exploration, providing invaluable scientific assets. Recent updates have resulted in a 20% increase in content in various categories, such as chemicals, genes, phenotypes, diseases, and exposure statements. A new feature called CTD Tetramers has been introduced, which constructs potential molecular pathways by linking a chemical, gene, phenotype, and disease. Furthermore, enhancements to the visuals of the webpage, including the integration of terms for human biological media and the corresponding anatomy pages, aim to improve the user experience and facilitate the exploration of environmental biomarkers in different tissues (Davis et al. 2023). These developments reaffirm CTD’s position as a practical, user-friendly, and innovative resource for accessing information and generating hypotheses about environmental health.

#### PubChem

PubChem, managed by the National Center for Biotechnology Information (NCBI), is the paramount repository of freely accessible chemical data worldwide. It was established in 2004 under the US National Institutes of Health’s Molecular Libraries Roadmap Initiatives and serves as a comprehensive public repository for chemical substances and their biological activities. It consists of three interconnected databases: Substance, Compound, and BioAssay. The substance database aggregates chemical information from diverse sources, while the compound database houses unique chemical structures extracted from substance data. The BioAssay contains biological activity data obtained from assay experiments (Kim et al. 2016). PubChem houses a wealth of invaluable information within its extensive archives, including chemical names, molecular formulas, structures, and a host of identifiers. This comprehensive database is a gateway to numerous chemical and physical properties, biological activities, safety profiles, patents, literature citations, and much more, spanning millions of compounds. Beyond merely cataloguing chemical compounds, PubChem goes the extra mile to streamline users’ access to critical safety information. Consolidated GHS classifications for more than 4000 substances, drawn from four reputable references, are readily available, providing users with a consolidated document that contains precautionary and hazard statements. This unified approach improves safety protocols by providing a single authoritative source for hazard classification. Moreover, PubChem acknowledges the paramount importance of laboratory safety. In alignment with the guidelines delineated in the National Research Council’s seminal publication, “Prudent Practices in the Laboratory: Handling and Management of Chemical Hazards” (2011) (National Research Council 2011), PubChem offers Laboratory Chemical Safety Summaries (LCSS). These summaries are invaluable, furnishing pertinent chemical hazard and safety information along with PubChem compound records and associated GHS classifications. By integrating LCSS with its extensive database, PubChem ensures that researchers, educators, and practitioners can navigate chemical safety with confidence and efficiency. In recent years, PubChem has undergone substantial improvements. More than 120 new data sources have been integrated, including Google Patents data, expanding its patent collection. New data collections such as Cell Lines and Taxonomy facilitate convenient access to chemical information based on cell lines or taxa. Additionally, updates to the bioassay data model and enhancements to PubChem’s programmatic access protocols, PUG-REST and PUG-View, enable target-centric data download and provide a ‘standardise’ option for returning standardised chemical structures. PubChemRDF has also been significantly updated (Kim et al. 2023).

#### TOXBASE

TOXBASE, the UK’s National Poisons Information Service database, has been the primary resource for healthcare professionals seeking poison information since 1998 (Bateman 2002). It offers guidance on more than 21,000 substances and products, accessible online for registered NHS users and available by subscription for non-NHS entities. TOXBASE offers single-site subscriptions for access by all staff within one department, like an Emergency Department or Poisons Information Centre. Multiple-site subscriptions are also available for broader access, such as all units within a hospital or all poison centres within a region or country. To request a formal quotation for a subscription, users can fill out the form provided. Terms and Conditions for TOXBASE users are available for reference. Within the clinical areas of the UK’s NHS, TOXBASE is offered free of charge, with NHS users directed to register through the registration form on the homepage. The TOXBASE app is free for individual users with NHS email addresses. It provides rapid access to clinical information, including dosing and management advice. Feedback on content is encouraged, with entries authored by NPIS and regularly reviewed. Registered users can seek training advice from NPIS via email. A 24-hour telephone helpline is also available for complex cases, allowing direct consultation with NPIS clinical toxicologists.

### Databases and sources useful for cosmetic products

#### Cosmetic ingredient review (CIR)

Cosmetic Ingredient Review (CIR) is an independent nonprofit programme established in 1976 by the industry trade association, originally known as the Cosmetic, Toiletry and Fragrance Association and now known as the Personal Care Products Council (CIR 2024). This initiative was supported by the U.S. Food and Drug Administration (FDA) and the Consumer Federation of America, reflecting a broad base of support beyond the cosmetic industry itself. As the only programme of its kind globally, CIR operates in an open forum, providing a transparent platform for assessing the safety of ingredients used in personal care products. The program’s cornerstone is its rigorous scientific evaluations conducted by the Expert Panel for Cosmetic Ingredient Safety, independent of the industry trade association that funds it. This panel comprises professionals from various fields, including dermatology, toxicology, and consumer safety, who ensure a multidisciplinary approach to ingredient safety assessment. Since its inception, the CIR has reviewed more than 4740 individual cosmetic ingredients, determining that 4611 of these are safe for use under specified conditions and identifying others as unsafe (Boyer et al. 2017). The CIR operates under a robust set of procedures, overseen by a seven-member steering committee. This committee ensures that the programme adheres to its policy and direction, balancing representation from different stakeholders. The President and CEO of the Council chair the committee. It includes members such as a dermatologist representing the American Academy of Dermatology, a toxicologist from the Society of Toxicology, a consumer representative from the Consumer Federation of America, an industry scientist who also chairs the Council’s CIR Committee, the Chair of the Expert Panel and the Council’s Executive Vice President for Science. Regular updates and rereviews of ingredients are essential aspects of CIR procedures, incorporating the latest scientific data to maintain the relevance and accuracy of safety assessments. These ongoing efforts are vital, as they provide crucial assurances about the safety of cosmetic ingredients, thereby benefiting both consumers and manufacturers. Through this open and scientifically rigorous process, the CIR helps to foster trust and ensure the safety of personal care products on the market.

It should be underlined that the transcripts of panel discussions conducted by the CIR serve as invaluable sources of information. They provide detailed insights into the criteria and arguments presented by the experts during the review process. This transparency in the exchange of information underscores the importance of these documents, as they reveal the scientific and regulatory considerations that influence the evaluation of cosmetic ingredients. Access to such detailed discussions enhances understanding of the methodologies and safety standards employed by the panel, making these transcripts a critical resource for anyone interested in the specifics of cosmetic ingredient safety evaluations, especially safety assessors.

#### Cosmetic ingredients (CosIng)

CosIng, the European Commission database (Bravo et al. 2020), is an authoritative resource providing comprehensive information on cosmetic substances and ingredients as defined in the Cosmetics Regulation (EC) No 1223/2009 and its precursor, the Cosmetics Directive 76/768/EEC (European Commission 2026). Since the 1976 adoption of the Cosmetics Directive, this database spans data, categorising entries into ‘active’ for current data and ‘not active’ for historical records. It is designed to assist with the regulatory compliance and labelling of cosmetic products in the European market. The database includes a glossary of common ingredient names essential for product labelling, as established by Decision (EU) 2019/701, and integrates opinions from the Scientific Committee for Consumer Safety (SCCS) on cosmetic ingredients. Additionally, CosIng enables searches for CAS, ELINCS, or EINECS numbers, providing a linkage to the chemical identity of substances. In CosIng, the differentiation between ‘ingredients’ and’substances’ is marked: Ingredients, listed in capital letters with an “I” symbol, are included for labelling purposes and do not imply authorisation. In contrast, substances are designated in small letters with an “S” symbol, referring to chemical elements and compounds regulated by the Cosmetics Regulation. This distinction underscores that while the CosIng database serves an informational purpose, only the Cosmetics Regulation carries legal authority on ingredient safety and regulatory compliance. 

#### International fragrance association (IFRA)

The IFRA database (International Fragrance Association) database is an essential resource that provides comprehensive information on the safety and regulatory status of fragrance ingredients used in consumer products, particularly cosmetics (https://ifrafragrance.org/) (International Fragrance Association 2024). It includes detailed safety assessments, guidelines on the permissible use of various ingredients, data on typical concentrations, and specifics on potential allergens. This database is crucial to ensure that cosmetic products are safe for consumer use and comply with international standards. One of the primary reasons the IFRA database is important is its focus on consumer safety. By providing thorough safety evaluations and setting regulations for the use of ingredients, the IFRA helps mitigate the health risks associated with cosmetics. It also helps manufacturers adhere to global safety standards, ensuring that their products can be safely sold in international markets. The IFRA approach to safety evaluation is built on the foundation of scientific rigour and transparency. IFRA provides a valuable resource through its Standards Library, a comprehensive, concise, sortable, and searchable database. This tool contains all the IFRA standards, allowing manufacturers, researchers, and the public easy access to understand and comply with the safety requirements for fragrance materials. The availability of this database further emphasises the commitment of the IFRA to transparency and its role in promoting the safe and informed use of fragrance ingredients across the industry. The IFRA Standards Library provides detailed safety and usage guidelines for fragrance materials, including chemical identification, implementation deadlines, and maximum acceptable concentrations for various product categories. Each document in the library outlines the scientific basis for the safety recommendations, supported by references to relevant safety assessments and research. Additionally, the conclusions of the Expert Panel for Fragrance Safety are detailed, explaining the rationale behind the established safety limits. This comprehensive resource ensures that manufacturers and stakeholders have access to crucial information for a compliant and safe product formulation. A critical aspect of the role of IFRA is especially allergen management. Fragrance allergens can trigger adverse reactions, such as contact dermatitis, among sensitive individuals. The database identifies these allergens and provides guidelines on their levels of safe use, helping formulators minimise the risk of allergic reactions and improve product safety. 

#### Research Institute for Fragrance Materials (RIFM)

The Research Institute for Fragrance Materials (RIFM) plays an essential role in the safety assessment of fragrance ingredients through its databases and publications (RIFM 2024). RIFM has established a collaboration with Elsevier to form the Food and Chemical Toxicology Fragrance Material Safety Assessment Center (RIFM 2024). This platform provides open access to peer-reviewed safety assessments and associated research, ensuring transparency and accessibility to data that support the safe use of fragrance materials.

The approach of RIFM to safety evaluations is based on strict guidelines detailed in the ‘Criteria Document’ (Api et al. 2015), which outlines the methodology for conducting safety evaluations. This document is crucial in integrating the latest scientific methodologies and testing strategies to ensure that fragrance ingredients are evaluated with precision and scientific rigour. The RIFM database features current Safety Assessments and offers a historical data section. This section includes group studies and background studies that predate the implementation of the Criteria Document, providing a comprehensive archive for researchers and industry stakeholders. RIFM’s databases are essential for protecting consumers and the environment in fragrance material use by facilitating easy access to current and historical safety evaluations.

#### Scientific committee on consumer safety (SCCS)

The Scientific Committee on Consumer Safety (SCCS) provides scientific advice to the European Commission on the safety of cosmetic products and cosmetic ingredients in the European Union (European Commission 2026).

SCCS opinions are compiled on the European Commission website in a structured repository organised by opinion status and topic (European Commission 2026). The repository includes “Preliminary Opinions open for comments”, described as opinions developed on the basis of specific sets of data (dossiers) submitted by applicants—for example, industry or Member State authorities—to satisfy regulatory requirements; these preliminary opinions are made available for clarification and comment for a minimum period of four weeks and, if possible, eight weeks, with an explicit timeline provided for each opinion (European Commission 2026). The same repository also lists “Opinions being finalised”, where the commenting period has expired and received comments are discussed in working groups and plenenary meetings with a view to finalising the opinions, and “Final Opinions”, which are presented under topic headings including Colorant, Fragrance, Hair dyes, Nanomaterial in cosmetic ingredients, Preservatives, UV Filters, and Other (European Commission 2026). Individual entries in the repository are provided as downloadable documents and are accompanied by basic identifiers such as an SCCS reference number and adoption date; selected entries also include notes on adopted corrigenda (European Commission 2026).

#### Cosmetic Europe

Cosmetics Europe is the main trade association representing the cosmetics and personal care industry in Europe (Cosmetics Europe 2024). Established more than 60 years ago, it serves as the unified voice for companies and national associations within this sector throughout the continent. This organisation plays a crucial role in interfacing with policymakers to shape regulations that are both effective and conducive to industry growth, ensuring that the industry’s perspective is considered when formulating European regulatory policies.

Cosmetics Europe’s mission is to foster a regulatory environment that supports longterm growth and sustainability for the cosmetics and personal care industry. Its vision is centred on a thriving sector capable of continuous innovation and expansion. Membership in Cosmetics Europe enables entities to engage directly with regulatory developments, influencing the legislative landscape that impacts the industry.

Cosmetics Europe maintains a library of resources, including detailed guidelines on the Cosmetics Directive, which clarify product safety, quality, and claims. Documents such as the “EU Cosmetics Directive Explanatory Brochure” elucidate the background, legislative process, and objectives of the directive, helping members and stakeholders navigate the complex regulatory environment.

In addition, the “News & Events” section of the Cosmetics Europe platform highlights the latest developments and upcoming events relevant to the cosmetics industry. This ensures that all members can access current information and participate in industry-shaping discussions and forums. This proactive approach to communication underscores the association’s commitment to leading the industry forward in a dynamic regulatory landscape.

Cosmetics Europe has embarked on a significant initiative to advance regulatory-approved animal-free testing methods for safety assessments. As part of this initiative, they have developed a database to support the industry’s transition to non-animal testing. This comprehensive database contains information on 128 substances and integrates five selected non-animal test methods for skin sensitisation. DPRA (Direct Peptide Reactivity Assay), KeratinoSens™, h-CLAT (human cell line activation test), U-SENS™, and SENS-IS. This database includes existing data and incorporates new data, comprising one-third of all information. These data are enhanced with human and local lymph node assay (LLNA) reference data and detailed information on the physicochemical properties and use categories of the substances. The selection of substances, although focused on the availability of human data, shows a wide diversity in chemical properties and use categories (Hoffmann et al. 2018).

#### Integrated in silico models for the prediction of human repeated dose toxicity of cosmetics to optimise safety (COSMOS)

COSMOS (Cosmetics Safety Modelling Of Substances) was an integral project within the SEURAT-1 group, a European research initiative to replace animal testing in safety evaluations with alternative methods (MNAM 2024). From January 2011 to December 2015, COSMOS focused on developing non-animal-based tools and workflows to enhance the assessment of cosmetic ingredients’ safety for human use. The COSMOS Database (DB), originally part of the SEURAT-1 initiative, has evolved into COSMOS Next Generation (NG) (Yang et al. 2021). This advanced platform combines a database with in silico tools, forming a comprehensive knowledge base essential for the cosmetics industry. The COSMOS DB provided a detailed inventory of cosmetics-related chemicals, including regulatory inventories and toxicity data from both in vitro and in vivo studies. Significant efforts in data curation and governance were made to ensure the quality and reliability of the data, including data authorisation, quality characterisation, metadata documentation and controlled data use. This rigorous management enabled the integration of various types of data from various sources. Based on these foundations, COSMOS NG expanded the Minimum Inclusion Criteria (MINIS) for toxicity databases to enhance the reliability of the studies. COSMOS NG now features advanced analytical tools such as multiple chemical fingerprints to analyse structural similarity and new computational tools to predict molecular properties and identify chemical risks such as DNA and protein binding, liver toxicity, and genotoxicity. These capabilities facilitate complex tasks like chemical category formation and read-across, including a guided workflow that helps users predict a No Observed Adverse Effect Level (NOAEL) confidence interval for a target structure.

#### EDETOX

The EDETOX database, developed under the EDETOX programme, is a significant academic and regulatory resource dedicated to understanding the dermal absorption of toxic chemicals (Newcastle University 2016). This three-year research program, initiated on January 1, 2001, and concluding with a final report in 2005, was funded by the European Union’s Framework V under the Quality of Life, Environment, and Health Key Action Funding (project number QLKA-2000-00196) (Williams 2004).

The primary objectives of the EDETOX programme were multifaceted. The project aimed to evaluate the efficacy of in vitro models in several European centres to determine their relevance in the generation of data on the percutaneous penetration of chemicals. It also assessed the reliability and variation between laboratories to establish standards for these models. Another key goal was to improve the understanding of the processes of dermal absorption for significant environmental and occupational contaminants that impact human health, particularly where skin penetration could significantly contribute to total internal exposure.

Furthermore, the EDETOX programme sought to use in vitro and in vivo data generated within the project to refine and expand existing quantitative structure-activity relationship (QSAR) and physiologically based pharmacokinetic (PBPK) models. These models aim to predict dermal penetrant absorption rates and distribution more accurately.

A critical aspect of the project was the development of validated in vitro experimental strategies and the corresponding computational models to quantitatively measure the dermal absorption of chemicals. This initiative aimed to reduce the dependence on animal testing in risk assessment procedures related to skin exposure.

The EDETOX database itself, which remains freely available, was specifically crafted by Sarah Soyei and Faith Williams at Newcastle University. Provides a comprehensive collection of quantitative data for dermal exposure risk assessments. This includes information that aids regulatory authorities in progressing toward the assignment of quantitative skin notations for potentially hazardous chemicals. Today, the completion of the project has been documented, and a summary of the final report and a list of publications can be downloaded, providing invaluable resources for ongoing and future research in toxicology and dermal absorption.

### Databases useful for pharmaceuticals/drugs

Electronic databases have significantly transformed the landscape of toxicology by providing structured and accessible repositories of crucial information for drug safety evaluations. As regulatory bodies, such as the FDA, emphasise efficient review of substances, these databases have become essential tools. They compile vast amounts of toxicological data, which facilitate the identification of biologically active compounds and enhance safety assessments through advanced computational models and analyses (Arvidson 2008). The value of drug databases in toxicology is not only in their ability to store vast amounts of data but also in how they highlight adverse drug reactions (ADRs), which are particularly interesting to pharmacologists. Understanding ADRs is crucial to ensuring drug safety and effectiveness; databases provide comprehensive information on these reactions. Databases such as DrugBank are instrumental in this regard. They provide detailed drug-target interactions and comprehensive data on pharmacokinetics and pharmacodynamics, including potential ADRs. This information helps pharmacologists predict and manage the risks associated with drug therapies, ensuring patient safety (Knox et al. 2011).

#### DrugBank

The DrugBank database has become indispensable for researchers in pharmacology, medicine, and bioinformatics. It is a comprehensive repository for detailed drug data, linking chemical properties with biological targets and facilitating a wide range of applications from drug discovery to pharmaceutical education. Initially launched in 2006, DrugBank has been continually updated to expand its content and improve its utility for various applications, including in silico drug target discovery, drug design, and drug interaction predictions. The database integrates drug information with extensive target (protein) data, offering a unique resource that combines chemical, pharmacological, and toxicological data with annotations on drug targets and actions. This integration supports various research activities, such as drug metabolism and interaction prediction, making DrugBank a vital resource in drug research and development (Wishart et al. 2006). During the past two decades, DrugBank has undergone a series of significant updates to enhance and expand its database, which is essential for researchers and healthcare professionals. Initially focused on basic drug information, updates have progressively incorporated advanced data sets, including pharmacogenomic data, which helps to understand how genetic variations affect drug response. The inclusion of drug metabolism data has also been pivotal in providing insights into how drugs are processed in the body. This information is critical for determining doses and understanding potential side effects. Furthermore, DrugBank has expanded to include comprehensive ADMET profiles detailing drug absorption, distribution, metabolism, excretion, and toxicity. These profiles are crucial for drug development and safety assessments. DrugBank 5.0, released in 2018, significantly improved the database’s capabilities. It included more investigational drug data, crucial for developing new therapeutics and understanding their potential benefits and risks before they are available on the market. Additionally, this update brought new drug-drug interaction data, an essential component for managing multidrug regimens and avoiding harmful interactions. Furthermore, the update expanded the types of available pharmacoomic data, including pharmacogenomics, pharmacoproteomics, and pharmacometabolomics. These additions help predict drug efficacy and safety by tailoring personalised medicine approaches. Much drug spectral information was added, including mass spectrometry (MS) and nuclear magnetic resonance (NMR) data. This information is vital for the chemical analysis of drug compounds, further verifying the purity and composition of the drug (Wishart et al. 2018). With its latest release, DrugBank 6.0, the database includes more than 11,891 drugs, significantly expanding the quantity and quality of its data offerings. These include FDA-approved drugs, investigational drugs, extensive drug-drug interactions, and drug-food interactions. The database has also seen significant improvements in experimental and predictive spectral data, which further supports pharmaceutical research (Knox et al. 2024). DrugBank is a valuable resource for pharmacology and toxicology due to its comprehensive “Toxicity” section. This database segment provides detailed information on the potentially toxic effects of various drugs and the available treatment options. Importantly, all of the information is supported by citations from the scientific literature, making it a reliable source for researchers and practitioners in toxicology.

#### European medicines agency (EMA) database

The European Medicines Agency (EMA) is an essential entity within the European Union dedicated to scientifically evaluating and supervising medicines for human and veterinary use. Established in 1995 and located in Amsterdam, the EMA plays a critical role in ensuring public and animal health by authorising and monitoring medicines throughout the EU and European Economic Area (EEA). The agency facilitates access to medicines by evaluating applications for marketing authorisation, monitoring safety throughout a medicine’s lifecycle, and providing crucial information to healthcare professionals and patients. Once approved, this centralised process allows companies to market their products across all EU and EEA countries, which is particularly significant for innovative medicines. EMA’s operations include the development of scientific guidelines and the provision of scientific advice, which supports the development of new medicines, particularly those for children and rare diseases (EMA 2024; Permanand et al. 2006; Teixeira et al. 2020).

EMA provides a section called Medicines on its website. This section offers a comprehensive over-view of medicinal products, including a searchable database of medications. Each entry typically includes detailed information about the medicine, such as its active ingredients, therapeutic uses, details of the marketing authorisation, and a link to the assessment report, if available. A typical final assessment report by the EMA comprehensively evaluates medicinal products. It begins with an introduction that sets out the background and objectives of the evaluation, including its legal basis. Nonclinical and clinical data sections summarise the pharmacological, toxicological, and clinical safety data collected along with efficacy studies. Pharmacovigilance aspects are reviewed, noting any adverse effects observed during the use of the product. Each section aims to outline the scientific evidence supporting the product’s safety and efficacy. The report concludes with an overall benefit-risk assessment, which weighs therapeutic benefits against potential risks, guiding the regulatory decision-making process. This structure ensures a thorough review and aids to maintain high standards for health products in the market. The report also contains many valuable pieces of information from a toxicological perspective, all based on extensive scientific research. The included sections such as single-dose toxicity, repeat-dose toxicity, genotoxicity, carcinogenicity, reproductive and developmental toxicity, and local tolerance provide detailed insights into the safety profile of the substance under review (EMA 2024).

#### FDA’s adverse event reporting system (FAERS)

The FDA Adverse Event Reporting System (FAERS) is a critical resource maintained by the U.S. Food and Drug Administration (FDA) for tracking adverse events and medication errors linked to the use of drugs and biological products after they are approved. The main components of FAERS include reports of adverse events, medication errors, and product quality complaints that could result in adverse effects. Healthcare professionals, consumers, and manufacturers voluntarily submit these reports to the FDA. One of the primary limitations is the uncertainty regarding causality. The system collects data on adverse events associated with drug use, but these reports do not confirm whether the drug caused the event. This means that events could be related to other factors, such as underlying conditions or medications taken concurrently, or could be coincidental. Another significant limitation is the completeness and precision of the reports. Many reports may lack sufficient detail to fully assess and understand the event. In addition, there is a risk of duplicate reports in which the same event is reported more than once, which can skew the data. Furthermore, because reporting is voluntary, there is inherent bias due to underreporting. Events are often not reported if they are perceived as minor or if the drugs involved have been on the market for a long time and are well established. Despite these limitations, FAERS data is accessible to the public through various means, enhancing transparency and facilitating further research. The FAERS dashboard offers an interactive platform for querying data, making it userfriendly and more approachable for individuals without a technical background. For those with an expertise in data management, the raw data files can be used to create more detailed relational databases. Additionally, individual case safety reports can be requested through Freedom of Information (FOI) requests directly from the FDA, providing another layer of detailed data for researchers. Understanding these limitations and access methods is crucial to effectively using the FAERS database for research, clinical decision making, or potential risks associated with certain medications (U.S. FDA 2019).

#### FDAble

FDAble is a unique initiative focused on enhancing medication safety by making it easier for individuals and professionals to access and analyse data from the FDA’s Adverse Event Reporting System (FAERS). Comprising a team of dedicated individuals passionate about public health and safety, FDAble aims to disseminate crucial medical information to prevent adverse drug events and improve patient out-comes. Believing that increased accessibility to FAERS data can lead to substantial improvements in medication safety, FDAble believes that open access to drug safety data is the key to fostering innovation in how these data are searched, mined and analysed. Their platform serves as the only publicly available search engine specifically designed to explore FAERS / AERS data, allowing users to dig into detailed reports of drug-related adverse events (U.S. FDA 2024).

#### RxList.com

RxList.com is a web-based medical resource that provides up-to-date pharmaceutical information about branded and generic medications. Established in 1995 by pharmacists, it has become the leading resource for the Internet Drug Index. In 2004, RxList was acquired by WebMD, enhancing the platform with contributions from a team of knowledgeable pharmacists and physicians. The content is regularly updated through collaborations with respected entities such as the FDA, Cerner Multum, and First Databank, Inc., ensuring the reliability and accuracy of the information provided. Users can search for drug information on RxList through the Drugs A - Z list, which includes both brand and generic names, or by typing the name of the drug in the search box. The website also features a pill identification tool for additional assistance (WebMD 2024).

RxList is a comprehensive online drug information database widely used in healthcare for clinical decision support. It offers detailed information about various medications, including their uses, side effects, and interactions, which are essential for safe prescribing and medication management. Clauson et al. (2007) evaluated several online drug information databases and found that while RxList is commonly used, it was lower than others in completeness and ease of use. Despite this, RxList remains a popular choice, possibly due to its accessibility and comprehensive drug listings (Clauson et al. 2007). Moreover, another aspect of RxList’s utility is its ability to support the identification of solid oral dosage forms through imprint codes as explored by Raschke et al. While RxList is part of the suite of tools used for this purpose, it performs similarly to other databases in the identification of drugs based on physical characteristics (Raschke et al. 2003).

#### Electronic medicines compendium (eMC)

The Electronic Medicines Compendium (eMC) is a critical resource in toxicology because it provides comprehensive and updated information on adverse drug reactions. Using advanced natural language processing techniques, the eMC facilitates the automatic extraction of critical data from detailed product summaries, which are crucial documents containing safety information required by regulatory authorities (Shen and Spruit 2021). In the eMC, each medication’s profile includes a section on adverse drug reactions, which lists potential side effects and toxic responses observed during clinical trials or reported post-marketing. The compendium goes beyond mere listings; it provides frequencies of these reactions and, in some cases, discusses the pathophysiological mechanisms behind them. This level of detail helps healthcare professionals make informed decisions about risk versus benefit when prescribing these medications to patients with complex medical histories. In addition, the availability of these data is beneficial in the broader context of public health monitoring and drug safety surveillance. By aggregating and analysing data on adverse drug reactions, researchers and regulators can spot patterns that may indicate underlying problems with drug formulations or reveal interactions with other substances that were previously unknown. This analysis can lead to updates in clinical guidelines and, if necessary, reevaluating a drug’s market authorisation. Therefore, the eMC serves as a static repository of drug information and a dynamic tool that supports ongoing pharmacovigilance and toxicological evaluation. Its integration of regulatory data ensures that all information reflects the most current standards and recommendations, which is crucial for maintaining patient safety in an ever evolving healthcare landscape (Ursu et al. 2017).

## Toxicological databases important for forensic toxicology

Forensic toxicology is a crucial discipline in forensic science. It is charged with identifying and analysing toxic substances in biological specimens to determine their role in criminal investigations, civil cases, and public health concerns (Ibrahim and Hassan 2022). In pursuit of these objectives, forensic toxicologists rely heavily on access to comprehensive databases containing information on the properties, effects, and toxicity of various chemical substances. In the field of forensic toxicology, the most important substances include those related to intoxication both for living and deceased people, postmortem. The greatest threat appears to be the growing interest in new psychoactive substances (NPS), among which new substances differing in their substituent are constantly being recognised (Favretto et al. 2013; Krotulski et al. 2024). Other than that, drug-facilitated sexual assault (DFSA) cases are important from a forensic toxicology point of view (Anderson et al. 2017). Rapid evaluation of sedative substances used might be crucial in the criminal court process. The im-portance of forensic toxicology is not only limited to previous examples, as it also incorporates cases of doping in sports and driving under the influence of psychoactive substances (Blandino et al. 2022) and others (Davies et al. 2016). The matrices used play a crucial role in proper identification of substances due to the concept of ADME (Absorption, Distribution, Metabolism and Excretion), which indicates that various metabolites of given substances might be found in different systems and organs (Vrbanac and Slauter 2017). Alternative matrices are crucial in forensic toxicology due to their unique benefits and potential use when using blood or urine samples (de Campos et al. 2022). For example, hair analysis can provide a historical record of drug use over a long period, offering valuable information for investigations involving chronic substance abuse (Pragst and Balikova 2006).

Toxicological databases help to interpret toxicological findings by providing context for the observed effects of substances on the human body. Understanding the mechanisms of toxicity, dose-response relationships, and physiological effects of various compounds is essential to deter-mine their relevance to a particular case (Borgert et al. 2021). By consulting these databases, forensic toxicologists can correlate the observed symptoms or pathological findings with the known toxic properties of specific substances, helping to establish the causality and contributing to the general determination of the manner and cause of death. Furthermore, toxicological databases facilitate the comparison of analytical results across different laboratories and jurisdictions. Standardised reference data on substance concentrations, detection limits, and analytical methods ensure consistency and reliability in forensic toxicology testing. Data harmonisation is essential for establishing scientific validity and credibility in legal proceedings, where accurate and reproducible results are para-mount.

### CFSRE: the center for forensic science research and education

The Center for Forensic Science Research and Education (CFSRE) (official online site: https://www.cfsre.org) was founded in 2010 in Willow Grove, Pennsylvania, under the Fredric Rieders Family Foundation. Its primary objective is to improve the proficiency, recognition and credibility of forensic science through training, education, and research. In particular, its NPS Discovery programme detects illicit drug markets to identify new substances in street drugs and distributes information about them (Mohr et al. 2022). CFSRE collaborates with various institutions to document the emergence of NPS in the US and globally. Through comprehensive analytical techniques, they generate “new drug monographs” containing data and information about these substances, such as crucial characteristics and available spectra of various analytical techniques (e.g. GC-MS, LC-QTOF-MS). Although not externally peer reviewed, the monographs undergo internal review by scientific experts.

In addition to that, CFSRE’s NPS Discovery provides trend reports on NPS presence in the US, aiming to offer up-to-date data on prevalence, positivity, and turnover. These reports use toxicology samples and drug materials.

In terms of forensic toxicology, CFSRE provides resources focussing on drug-impaired driving, drug-involved death, drug-facilitated crimes, and ultraesoteric toxicology testing (Fiorentin and Logan 2019; Geraldo de Campos et al. 2020).

### Erowid

The main focus of Erowid (available online: https://erowid.org/) is to provide access to unbiased information on psychoactive substances and related topics. They collaborate with experts to develop and enhance resources while ensuring their preservation for the future. Their vision is of a world where psychoactives are approached with respect and understanding, fostering informed decision-making and healthier behaviours. Erowid aims to contribute to this vision by promoting truth and integrity in the dissemination of information on psychoactivity (Erowid 2024; Mooseder et al. 2022).

### DrugsData.org (formerly EcstasyData)

DrugsData (available online: https://www.drugsdata.org/index.php), launched in July 2001, is an independent laboratory drug analysis programme affiliated with the Erowid Centre. It collects, manages, reviews, and presents drug analysis results publicly accessible to aid harm reduction, medical professionals, and researchers. Although it gathers results from various sources, it primarily conducts its tests through Drug Detection Labs (DDL). Samples are submitted anonymously by mail for testing (Erowid 2016).

### National forensic laboratory information system (NFLIS)

NFLIS (available online: https://www.nflis.deadiversion.usdoj.gov/) stands for the National Forensic Laboratory Information System. It is a programme administered by the Drug Enforcement Administration (DEA) in the United States. NFLIS is a nationwide database that collects and analyses forensic drug evidence from local, state, and federal laboratories throughout the country. Its primary objective is to provide accurate and timely information on drug trends, trafficking patterns, and the prevalence of various substances encountered in forensic investigations. This allows law enforcement agencies, public health officials, policymakers, and researchers to better understand trends in drug abuse and make informed decisions related to drug policy and enforcement efforts. NFLIS comprises three components: NFLIS-DRUG, NFLIS-TOX, and NFLIS-MEC. NFLIS-DRUG collects data from forensic laboratories nationwide on drug identification results from law enforcement cases. It helps to monitor illicit drug use and trafficking, including the diversion of pharmaceuticals to illegal markets. This component details the substances analysed, the purity, quantity, and drugs found in the same case, supporting drug scheduling decisions and enforcement initiatives. In 2019, the NFLIS programme expanded to include NFLIS-TOX, which gathers toxicology testing data from public and private laboratories for both postmortem and antemortem cases, and NFLIS-MEC, which collects information from medical examiner and coroner offices on deaths where drugs were identified. These additions complement NFLIS-DRUG and assist the DEA in drug regulation and scheduling efforts (Pitts et al. 2023).

### European monitoring centre for drugs and drug addiction (EMCDDA)

The EMCDDA (available online: https://www.emcdda.europa.eu/index_en) stands for the European Monitoring Centre for Drugs and Drug Addiction. It is the central hub for drug-related information within the European Union (EU). Its primary role is to provide the EU and its member states with accurate data on European drug issues and a unified framework for discussing drugs. The EMCDDA operates through a network of 30 national monitoring centres known as the Reitox National Focal Points. It publishes reports covering various drug-related topics, including epidemiology, interventions, laws, and policies. Annually the EMCDDA releases its Annual Report, offering updates on the European drug situation translated into various languages. Although not a formal research centre, the EMCDDA facilitates the interface between scientific findings and policymaking, sparking new research inquiries and guiding priority identification. The key challenges for the agency include maintaining high scientific standards while fulfilling its institutional role and helping European efforts to establish, share, and standardise drug best practices (Griffiths et al. 2012).

### HighResNPS

HighResNPS.com (https://highresnps.com), a crowd-sourced mass spectral database for HR-MS screening of NPS, is managed and supported by The International Association of Forensic Toxicologists (TIAFT). The libraries in various formats contain 2,323 compounds with fragment data of 1,658. Users can receive the database with the predicted retention times for more than 2,200 compounds. The main page displays consensus data for unique compounds, including “artificial” spectra for those with recorded product ions. The database supports direct searches for compound names and the exact mass of precursor and/or fragment ions and can be converted into suspect libraries for various mass spectrometry platforms. However, access to this database is restricted to registered users who could gain access through email requests (Mardal et al. 2019).

### Agency for toxic substances and disease registry (ATSDR)

The Agency for Toxic Substances and Disease Registry (ATSDR) is a federal public health agency within the United States Department of Health and Human Services (HHS) (El-Masri et al. 2002). Its primary role is to serve the public by using the best science, taking responsive public health actions, and providing reliable health information to prevent harmful exposures and diseases related to toxic substances. ATSDR works closely with communities, other federal agencies, state and local governments, and other stakeholders to accomplish its mission. It conducts research, assesses health risks from hazardous waste sites, responds to emergencies involving hazardous substances, and provides guidance and recommendations for protecting public health. ATSDR resources are available online: https://www.atsdr.cdc.gov/.

### The organization of scientific area committees for forensic science (OSAC)

The Organization of Scientific Area Committees for Forensic Science (OSAC) is a program established by the National Institute of Standards and Technology (NIST) and the Department of Justice (DOJ) in the United States. OSAC aims to strengthen the reliability and validity of forensic science by developing consensus-based standards and guidelines. OSAC operates through subcommittees and working groups, each focussing on specific forensic science disciplines such as DNA, toxicology, firearms and tool marks, digital evidence, etc. These groups consist of experts from various backgrounds, including forensic scientists, researchers, academics, and practitioners. By promoting collaboration and standardisation within the forensic science community, OSAC aims to improve the quality and consistency of forensic analysis, improve courtroom testimony, and ultimately increase confidence in the trust of the justice system in forensic evidence (Jones et al. 2023). The OSAC standards for forensic science are available online: https://www.nist.gov/organization-scientific-area-committees-forensic-science/osac-registry/.

## Toxicological databases important for food toxicology

### OpenFoodTox: chemical hazards database

In broad terms, this database is denoted as an openly accessible toxicological database with respect to chemical compounds in food and animal feed. OpenFoodTox, an open source toxicology database, is available for downloading and visualising data, offering information on all substances evaluated by the European Food Safety Authority (EFSA). It includes substance characterisation, links to EFSA findings, relevant regulations, and summarised data on hazard identification and characterisation risks to human health, animal health, and ecological evaluations (Dorne et al. 2021). The database has been developed using OECD standardised templates for reporting summaries of chemical tests (OHT) to facilitate data sharing with stakeholders interested in chemical risk assessment, such as related agencies, international scientific advisory bodies, etc. OpenFoodTox 2.0 includes properties such as physicochemical characteristics, exposure data, and toxicokinetic information. Additionally, plans exist to integrate in silico modelling platforms like QSAR (free software aplication offers search for experimental data, simulating metabolism and profiling the properties of chemical substances) and physiologically based kinetic models. These structured hazard data from in vivo, in vitro, and in silico sources offer diverse lines of evidence that can be merged, weighted, and harmonised using standardised weight-of-evidence methodologies. This helps to support the application of new approach methods (NAMs) in chemical risk assessment and reduction of animal testing (EFSA 2021). OpenTox can be used for data on the characterisation of chemicals, regulatory information, EFSA findings, toxicity assessments, reference points (NOAEL, BMD, LD50, etc.), reference values (ADI, TDI, PNEC, etc.), uncertainty factors and scientific results from EFSA (EFSA 2022). A solution to the different weights of evidence of individual data could be the introduction of the Cramer classification for oral exposure using the TTC RepDose database (Tluczkiewicz et al. 2011).

### Drugs and lactation database (LactMed^®^)

The LactMed^®^ database details medications and various substances that breastfeeding mothers may encounter. Provides information on the presence of these substances in breast milk and infant bloodstream, along with potential adverse effects on nursing infants. It also suggests alternative therapeutic options when applicable. All information is sourced from the scientific literature with comprehensive references and a peer review panel ensures its scientific accuracy and relevance (NIH 2006). This database was developed in 2006 (AHRQ 2006). The Database of Drugs and Lactation of the National Library of Medicine is an essential resource for any healthcare professional who treats or answers questions about breastfeeding women. It is available to everyone free of charge online through the TOXNET platform (Vaughn 2012).

### FoodData central (FDC)

FoodData Central (FDC) is the central hub for USDA-based food composition information, consolidating various data types into a single platform. Five distinct data categories within FDC offer comprehensive information on food and nutritional profiles. Among these categories, Foundation Foods (FF) and Experimental Foods (EF) stand out as pioneering resources, presenting previously unavailable data and metadata. The remaining three categories—Standard Reference (SR) Legacy, Food and Nutrient Database for Dietary Studies (FNDDS), and Global Branded Foods Products Database (GBFPD)—are familiar to many users. The USDA developed FDC to adapt to the changing landscape of food supply, analytical methods, and agricultural practices. This initiative addresses the growing demand for reliable, transparent and easily accessible nutritional and food ingredient information. FDC caters to diverse audiences, including public health professionals, researchers, policymakers, nutritionists, healthcare providers, product developers, and the general public, ensuring accessibility to meet the evolving needs of these interested parties.

### Evaluations of the joint FAO/WHO expert committee on food additives (JECFA)

This archive contains condensed overviews of the JECFA evaluations on flavours, food additives, contaminants, toxins, and veterinary medications. Each overview includes fundamental chemical details, ADIs/TDIs, references to recent reports and monographs, links to the specification database, and a timeline of JECFA assessments. The archive can be searched by partial name or CAS number, initial character (letter or symbol), or functional category (WHO 2024).

### Inventory of evaluations conducted by the joint meeting on pesticide residues (JMPR)

This compilation outlines the assessments conducted by the Joint FAO/WHO Meeting on Pesticide Residues (JMPR) regarding pesticides. It does not include the maximum residue levels (MRL) that JMPR recommends (WHO 2024).

### Global environment monitoring system (GEMS)/food contamination monitoring and assessment programme

Since 1976, the Global Environment Monitoring System - Food Contamination Monitoring and Assessment Programme, known as GEMS/Food, has provided information to governments, the Codex Alimentarius Commission, other pertinent bodies, and the public. Reports on the levels and patterns of food contaminants, their impact on human exposure, and their importance for public health and trade. This initiative is carried out by WHO in collaboration with more than 30 WHO Collaboration Centres and recognised national institutions worldwide (WHO 2024).

### FDA toxicity databases

The FDA (U.S. Food and Drug Administration) maintains several toxicity databases to assess the safety of food additives, drugs, and other substances. These databases contain information on the toxicological properties of various compounds, including their potential to cause adverse effects in humans and animals. One of the most well-known FDA toxicity databases is the Toxicology Data Network (TOXNET). TOXNET provides access to databases covering toxicology, hazardous chemicals, environmental health, and related areas. Some of the databases included in TOXNET are the Hazardous Substances Data Bank (HSDB), which contains comprehensive toxicology data for more than 5,000 chemicals; the Integrated Risk Information System (IRIS), which provides information on the health effects of environmental contaminants; and the Chemical Carcinogenesis Research Information System (CCRIS): Focusses on data related to the carcinogenicity of chemicals, Toxics Release Inventory (TRI): Offers data on the release of toxic chemicals into the environment. These databases serve as valuable resources for researchers, regulatory agencies, and healthcare professionals in assessing the potential risks associated with exposure to various substances. They are crucial in informing regulatory decisions and public health policies to protect human health and the environment (Arvidson 2008).

### FADB (food additives database)

FADB (Food Additives Database) is an appropriate and publicly available data collection on food additives. The FADB is a threedimensional version of the EAFUS (All Added to Food in the United States) list, the sum of the WHO and FAO food additive databases. It can provide helpful information in the initial stages of toxicological assessments. Molecules in FADB are represented by several chemical and 1D identifiers, physical properties, and 3D file formats (SD and Mol2 files). The FADB also contains important information on the functional uses of chemicals as food additives (Ginex et al. 2014).

### Global environment monitoring system: food contamination monitoring and assessment programme (GEMS/Food)

In fulfilment of its responsibility for dietary exposure evaluation, GEMS/Food has formulated supranational diet models that are currently employed to forecast the intake of various chemicals in diets, adhering to globally recognised methodologies (referenced in EHC 240). The GEMS/Food Cluster diets—2012 Dashboard showcases a world map and consumption statistics for each of the 17 GEMS/Cluster diets. Users can choose a group, which enables them to access information on the countries within that group and consumption figures in grammes per person per day for the selected group (WHO 2024).

### US food and drug administration’s (FDA) toxic elements in food and foodware and radionuclides in food compliance programme

This initiative aims to oversee products, explicitly focussing on foods and related items intended for food use, such as glazed ceramicware and silver-plated hollowware, which are likely to contribute to the dietary intake of harmful elements and radioactive substances. The Centre for Food Safety and Applied Nutrition (CFSAN), in collaboration with the Agency’s field personnel, is responsible for protecting public health by ensuring the safety of the food supply. Regular monitoring of chemical contaminants in foods and the potential intake of these contaminants is conducted. In particular, toxic elements and radionuclides are of particular concern; their presence in foods can originate from various sources, including historical agricultural practices (e.g., use of pesticides containing heavy metals), industrial discharge, nuclear weapons testing, nuclear power generation, and release of harmful elements from food-contact containers or utensils (C. for F. S. and A. U.S. FDA 2024).

### Pesticide data programme (PDP) of the US department of agriculture (USDA)

The Pesticide Data Programme (PDP) is a nationwide initiative that monitors pesticide residues, resulting in the most extensive pesticide residue database in the United States. The oversight of PDP activities, which include the sampling, testing, and reporting of pesticide residues on agricultural products within the US food supply, is managed by the Monitoring Programmes Division. Special attention is paid to commodities that are highly consumed by infants and children. This programme operates in collaboration with State agriculture departments and various federal agencies. The data collected through PDP serve several key purposes:

- they enable the U.S. Environmental Protection Agency to evaluate dietary exposure to pesticides;
- they support the global promotion of US agricultural products;
- they guide the U.S. Food and Drug Administration and other governmental entities, helping them make well-informed decisions (USDA 2024).

## Toxicological databases important for environmental toxicology and ecotoxicology

Environmental toxicology, a field that investigates the permeation of substances in the environment and their potential adverse effects on plants, animals, and the broader ecosystem (Pawlaczyk and Szynkowska-Jóźwik 2024), significantly emphasises the exposure of ecological receptors to human-made substances. Therefore, collecting data on chemical substances and specific species is not only important but crucial for ecotoxicological systems, serving as a fundamental starting point for any research in this field (Russom 2002). Numerous online resources, known for their reliability, provide valuable chemical details, including chemical structure, CAS registry numbers, production sources, and physicochemical properties. Similarly, online platforms offer information about particular species, including taxonomic classifications, life histories, and migration patterns. These data, sourced from trusted sources, are essential for researchers and risk evaluators to comprehend the potential hazards and exposure levels that substances can pose to aquatic and terrestrial wildlife.

### PHYSPROP and EPI suite

The Syracuse Research Corporation (SRC) offers several complimentary chemical databases and software programmes for property estimation. The PHYSPROP database contains information on the chemical structures, names, and physical properties of more than 41,000 chemical compounds (SRC 2024). These physical properties, sourced from various outlets, encompass experimental, estimated, and extrapolated values. This resource is included in the Environmental Protection Agency’s (EPA) EPI Suite™ package (US Epa 2024). EPI Suite v4.11, a standalone software, comprises tools for estimating physicochemical properties, environmental fate, and ecotoxicity toxicity. The predicted data provided by EPI Suite are derived for a subset of the panel content using batch processing functions available in the application. Web services for these estimation programmes are also available (Williams et al. 2017). The models embedded within the EPI Suite software are designed to estimate a range of parameters, including the octanol-water partition coefficient (Kow), atmospheric oxidation rate, Henry’s law constant, melting and boiling points, vapour pressure, water solubility, biodegradation rates, sorption coefficients, bioaccumulation factors, hydrolysis rates, and toxicity to aquatic organisms. Experimental data for fish biotransformation half-lives, bioaccumulation, and bioconcentration factors were sourced from the curated PHYSPROP database (Mansouri et al. 2018). The ECOSAR version 2.2 modelling system version 2.2 employs quantitative structure-activity relationships (QSAR) to predict the toxicity of individual chemical substances. QSARs establish a link between the structural characteristics of the chemical under examination and structurally similar analogues with available toxicity data. ECOSAR is part of the EPI Suite package (US Epa 2024). It requires specific physicochemical parameters as input, such as the octanol-water partition coefficient.

### NIST chemistry WebBook

The National Institute of Standards and Technology (NIST) provides access to a database (https://webbook.nist.gov/chemistry/) that contains thermochemical, thermophysical, and ion energetics data (Linstrom 2023). Users can conduct searches based on molecular formula, structure, CAS registry number, or chemical name through the user interface. Search results include comprehensive information about these parameters, along with chemical synonyms, thermochemistry, Henry’s Law constants, gas-phase ion energetics, ion aggregation, mass spectra, and vibrational and electronic spectra; pertinent references to data sources are also provided.

### Environmental residue effects database (ERED)

The United States Army Corps of Engineers maintains the Environmental Residue Effects Database (ERED). It compiles data on the accumulation of harmful substances in tissues (through tissue residues) and their impact on various endpoints, such as mortality, growth, and physiological and biochemical responses (ERED 2017). Additionally, the database includes information on the concentrations at which a low effect was observed (LOED) and the concentrations at which no effect was detected (NOED). Spanning literature published between 1966 and 2017, the database encompasses more than 14,282 entries extracted from approximately 990 publications involving more than 340 species (https://ered.el.erdc.dren.mil/). ERED specialises in sediment toxicity and the influence of contaminants in materials dredged during channel deepening on freshwater organisms. Its purpose is to evaluate the potential of contaminant concentrations in dredged sediments to induce undesirable adverse effects on aquatic organisms (Bridges and Lutz 1999). Although ERED was originally developed as a tool for the US Army Corps of Engineers to address the adverse effects of channel dredging, its utility extends to the investigation and management of sediment toxicity in broader contexts.

### ECOTOXicology (ECOTOX)

The Environmental Protection Agency (EPA) established the ECOTOXicology database (ECO-TOX), which served as a central repository for accessing and retrieving toxicity data sourced from the AQUIRE (aquatic life), PHYTOTOX (terrestrial plants), and TERRETOX (wildlife) databases (US Epa 2024). The ECOTOX Knowledgebase comprehensively represents the ecotoxicological tests published over recent decades. Consequently, data can depict trends in species distribution, chemicals, and biological effect measurements over time within ecotoxicology. Additionally, assessing the current data landscape enables the prioritisation of literature searches and data curation and the identification of data deficiencies for chemical and species groups. As of the latest update (as of March 13, 2024), ECOTOX contains 167,326 individual effect records derived from 54,475 peer-reviewed publications, covering over 12,934 chemicals and 13,915 aquatic and terrestrial species (US Epa 2024). Chemicals associated with the toxicity data in ECOTOX include various groups such as metals, pesticides, pharmaceuticals, and perfluoroalkyl and polyfluoroalkyl substances, among others (Russom 2002). The ECOTOX database encompasses data on 13,915 biological species, reflecting a certain degree of taxonomic imbalance evident in the ecotoxicological literature. In particular, a mere 100 out of 13,914 species contribute to 50% of the overall record count, while more than 9000 species are represented by data sourced from merely one or two references. Fish is the most prominent taxonomic group, with 25% of records (273,693) and over 13,000 references within ECOTOX. On the contrary, taxonomic categories, such as reptiles, mosses, and hornworts, have the scantest records and references. Variances in data availability between individual species are notable within these taxonomic groups. The nature of the studied and reported effects has evolved substantially over the decades of ecotoxicological research. Presently, publications encompass a broader spectrum of effects, including behavioural and transcriptomic parameters, along with the traditional endpoints of growth, development, reproduction, and mortality commonly documented in the historical literature (Olker et al. 2022). The ECOTOX database adapts to these changing trends by continuously integrating effect measurement terminologies into its controlled vocabulary. Currently, ECOTOX hosts data on 6070 distinct effect measurements reported by authors, encompassing various endpoints beyond the standard apical toxicity metrics. These encompass organ- and cellular-level effects, biochemical alterations, genetic impacts, chemical accumulation, and behavioural responses (Guerbet and Guyodo 2002). Mortality, growth, and reproduction constitute 47% of records, featured in 39%, 32%, and 12% of references. The subapical biochemical, cellular, and genetic effects are included in 28%, 9%, and 7% of references, collectively accounting for 20% of the records. The “Quick Search” functionality allows users to conduct searches based on primary taxonomic classifications, scientific and commonly used species names, general media classifications (e.g., terrestrial, aquatic), CAS Registry number, chemical name, observed effect, and publication year. For more intricate search strategies, the “Advanced Search” option offers approximately 15 encoded parameters within the database, covering aspects such as database update date, specific media classifications (e.g., hydroponic, freshwater, marine, soil), exposure type (e.g., diet, injection), and test location (e.g., field, laboratory). Data retrieval is available in both tabular report format and ASCII text file format, with complete citations provided for every data record. The ECOTOX database can be accessed entirely in ASCII text file format. Numerous tools and applications leverage data sourced from ECOTOX, including those focussing on SSDs (e.g., Web-ICE USEPA, Chemical Aquatic Fate and Effects [CAFÉ] by the National Oceanic and Atmospheric Administration), predicted no-effect concentrations, and threshold values (e.g., the ecological threshold of toxicological concern, EnviroTox, NORMAN), QSARs (e.g., ECOlogical Structure Activity Relationships [ECOSAR] predictive model, Toxicity Estimation Software Tool [TEST], Organisation for Economic Cooperation and Development’s [OECD] QSAR Toolbox, Assessment Tools for the Evaluation of Risk [ASTER]), bioaccumulation modelling and validation, as well as the development of Adverse Outcome Pathways (AOPs). Recent enhancements to the ECOTOX Knowledgebase streamline the identification and access of pertinent data for the applications outlined above, together with those delineated in subsequent sections (Olker et al. 2022). While the ECOTOX Knowledgebase remains instrumental in providing vital information to support ecotoxicity decisions, it serves a diverse audience comprising government, academic and industrial researchers alongside risk assessors. However, recognising the imperative to align with evolving trends in reported toxicological outcomes, comprehensive updates have been carried out, including a redesign of the user interface for web-based access to the Knowledgebase. These enhancements empower users to dive into and visualise toxicity data, offering various data out-put/visualisation alternatives, and incorporating standardised identifiers (e.g., DTXSIDs) to foster enhanced interoperability and reusability.

### Geographic information system (GIS)

Geographic Information Systems (GIS) have been applied in the context of risk assessment and exposure evaluation. They help identify suitable areas for pesticide application, pinpoint locations at increased risk of pesticide drift beyond target areas, and map the distribution of native plant species (Eccles et al. 2019). The Environmental Protection Agency of the United States has developed a GIS-based tool incorporating factors like wind speed, pesticide application rates, spatial distribution of agricultural fields, and others to calculate the likelihood of exposing nontarget areas to unintended chemical hazards. It also helps identify more sensitive plant species and determine potential sites for field studies or monitoring efforts (Pfleeger et al. 2006). Enhancements to ecological risk assessments for pesticide registration could involve using response data from species commonly found in high-risk areas instead of extrapolating test data from species not directly linked to areas with the highest exposure potential.

### EnviroTox

Developing robust novel methodologies for supervised and unsupervised approaches requires the use of existing information through curated and integrated datasets. An emerging methodology is the Ecological Toxicological Concern Threshold (ecoTTC), which can predict a conservative minimal toxicity value for chemicals with limited or no available information (Belanger et al. 2015). To construct the ecoTTC tool, a comprehensive and varied collection of environmental data from numerous sources was harmonised, characterised and analysed for quality to ensure efficient organisation and extraction of data. The EnviroTox database is a reliable, meticulously curated repository containing high-calibre toxicity studies conducted in aquatic environments, traceable to their primary sources (Connors et al. 2019). Pertinent details about specific chemical substances are intricately linked to each dataset, encompassing physicochemical attributes, chemical descriptors, and classifications based on the mechanism of action. Toxicity-related information is intricately associated with physicochemical attributes, mechanism-of-action classifications, and meticulously curated taxonomic data about organisms subjected to testing. EnviroTox v. 2.0.0 comprises 91,217 records from acute or chronic toxicity studies, representing 1563 species and 4016 unique CAS numbers of chemical substances (HESI 2021). Data on acute toxicity predominate, with most originating from acute toxicity studies for fish or invertebrates. The database includes 738 chemical substances for which acute toxicity data are available for algae, invertebrates, and fish are available; these data can be used to compare relative sensitivities among trophic groups. Individual trophic groups exhibit effect concentrations ranging from 10 orders of magnitude (amphibians) to 15 orders of magnitude (invertebrates); this range of effect concentrations represents a blend of species. Algae were the most sensitive trophic group, followed by invertebrates and fish (48.8%, 28.5%, and 22.8%, respectively). The EnviroTox platform also features three analytical tools: a predicted no-effect concentration calculator, an ecoTTC distribution tool, and a chemical toxicity distribution tool. Although the EnviroTox database and tools were originally developed to support ecoTTC analysis and development, they have broader applications in ecological risk assessment.

### CalEcotox

CalEcotox is a repository dedicated to furnishing the essential data for the assessment of ecological risks and is under the California Environmental Protection Agency (OEHHA 2024). Operating as a species-orientated relational database, it amalgamates physiological and ecological parameters with toxicity information spanning over 90 species encompassing mammals, birds, reptiles, and amphibians indigenous to California. CalEcotox compiles species-specific data concerning approximately 30 exposure variables (e.g., body mass, consumption patterns, seasonal behaviours, and population dynamics) commonly employed in gauging pollutant exposure (Donohoe et al. 2000). In addition to these exposure metrics, the CalEcotox repository incorporates toxicological data on the effects at the population level and individual level for the respective species, wherever available. All data encapsulated within CalEcotox have been meticulously sourced from publicly accessible scientific literature. Its scope is focused on species endemic to California and chemicals that pose a threat to the state. Currently, the database encompasses 9478 data points in various categories of wildlife, including amphibians (677), birds (3252), fish (97), mammals (2236), and reptiles (1115). In the Ca/Ecotox repository, data of individual species links chemical exposure to effects, such as dose-response relationships and/or tissue concentrations, including bioaccumulation and biotransfer data. Residue data is incorporated only when associated with specific exposure or effect data. The toxicological endpoints utilised in CalEcotox encompass Exposure Indicators (TOX-EXP IND), Mortality (TOX-MORT), Non-Reproductive Sublethal (TOX-Non-Repro-Sublethal), Population/Community (TOX-POP), and Reproduction (TOX-REPRO). With respect to pollutants, the focus is on compounds such as polycyclic aromatic hydrocarbons (PAHs), polychlorinated biphenyls (PCBs), metals, pesticides, and mixtures such as crude oil and sewage sludge. An interactive search tool facilitates data retrieval based on selected chemical substances or species from a drop-down list. Users are prompted to choose a chemical substance or exposure factor upon selection of species, resulting in a summary table summarising the query intersection. Users must filter the results by selecting a species if a chemical substance is initially chosen. The user interface streamlines options after selecting the initial species or chemical substances. For each species in the CalEcotox database, comprehensive summary reports are accessible, which include all pertinent information on chemical effects or exposure factors.

### Aggregated computational toxicology resource (ACToR)

ACToR (Aggregated Computational Toxicology Resource) is a centralised, searchable repository of data concerning environmental chemicals accessible online and allows queries by CASRN, name, or chemical structure. It encompasses chemical identifiers and structures, physicochemical values, in vitro assay data, in vivo toxicology data, source information and links. While it primarily covers chemicals of interest to environmental scientists and regulators, it also includes data on pharmaceutical compounds due to collaborative efforts of environmental and pharmaceutical toxicologists to advance predictive methods for human toxicity using in vitro and computational techniques based on comprehensive data sets for model construction and validation (Judson et al. 2012). Currently, ACToR aims to (1) facilitate access to information on health impacts and potential exposure to environmental chemicals, (2) identify gaps in understanding environmental chemical toxicology, and (3) provide a platform for model development to address deficiencies in environmental health risk data. It aggregates data from various sources, including previously inaccessible or poorly searchable data, such as previously unavailable animal studies on pesticides. Furthermore, there is a notable scarcity of toxicology data for many environmental chemicals, resulting in a “data gap.” ACToR addresses this gap by compiling toxicology data for the major classes of environmental chemicals, including those produced in significant quantities, as defined by the Toxic Substances Control Act. Analysis reveals that only approximately 25% of widely used environmental chemicals have substantial toxicology data (Judson et al. 2008). Computational toxicology, employing a combination of in vitro data, chemical information, and computer modelling, offers a promising approach to predict human health risks associated with environmental chemicals. The U.S. Environmental Protection Agency’s ToxCast programme is a key initiative in this field and was instrumental in the development of ACToR. Detailed accounts of ACToR’s role in ToxCast can be found elsewhere.

### Distributed structure searchable toxicity (DSSTox)

The EPA’s Distributed Structure-Searchable Toxicity (DSSTox) Database serves as the foundational repository for various publicly accessible applications, including the Computational Toxicology Dashboard, the Ecotoxicity Knowledgebase, the Chemical Exposure Knowledgebase, and others. The database contains more than one million substances, including chemical lists of interest for the EPA, federal agencies, states, tribes, industry and other stakeholders (Richard et al. 2006). DSSTox ensures accurate linkage of chemical structures to source substance identifiers, such as Chemical Abstract Service Registry Numbers (CAS RNs) and chemical names. This linkage enables robust association of chemicals with existing toxicity data, bioactivity data, and experimental chemical property data, facilitating the utilisation of this information in structure-based predictive modelling. EPA continuously enriches DSSTox with high-quality chemical and chemistry data, allowing direct user access and providing a reliable public resource (Richard and Williams 2002). Since its establishment in 2004, DSSTox has prioritised meticulous data curation efforts to rectify errors in chemical identifiers, aiming to ensure precise alignment of chemical structures with data crucial for assessing chemical risk. Initially curated with 7,000 chemicals, DSSTox has expanded by automatically loading segments from three public datasets. EPA Substance Registry Services (SRS), National Library of Medicine ChemID, and PubChem. However, this process was limited by the need for uniquely mapped identifiers (e.g., CAS RN, name, and structure) for each substance, rejecting content in conflicting identifiers within or across datasets. This rejection underscored the prevalence of contradictory and inaccurate substance structure ID mappings in the public domain, ranging from 12% (within EPA SRS) to 49% (across ChemID and PubChem).

## Conclusions

In this review, we comprehensively explore the landscape of toxicological databases, highlighting their pivotal role within regulatory toxicology. We identified the strengths and shortcomings of current systems and suggested actionable improvements to enhance their functionality and interoperability. These databases are indispensable in the design of regulatory frameworks and provide essential data for risk assessment, regulatory compliance, and chemical safety evaluation. This supports informed decision-making that safeguards public health. Types of resources used (suite of tools) are pointed in Table 1. 

Despite their extensive use, these databases often suffer from issues related to data accessibility, consistency, and integration. The disparity in database formats and the lack of standardised protocols hinder efficient data retrieval and cross-database searches, posing significant barriers to users. To bridge these gaps, we recommend improving standardisation by implementing uniform data standards across databases to facilitate smoother data integration and retrieval. Improving accessibility by redesigning user interfaces to be more intuitive and user-friendly is crucial, ensuring all potential users can navigate and use the databases effectively. Establishing protocols for periodic review and updating database content is necessary to keep up with scientific advances and regulatory changes. Table 2 provides a qualitative overview of commonly observed strengths and limitations of toxicological databases (it does not compare specific databases or regulatory regimes).

In looking to the future of toxicological databases, several key directions emerge that could profoundly enhance their utility and impact on public health and regulatory science. Integrating advanced technologies such as artificial intelligence (AI) and machine learning (ML) could revolutionise how toxicological data are processed, analysed, and interpreted. These technologies offer the potential to automate data curation and analysis, reduce human error, and provide deeper insights through predictive modelling and simulation. This could accelerate the pace of toxicological assessments and improve their accuracy and reliability.

Additionally, there is a pressing need to improve interoperability and standardisation between different toxicological databases. The development of global data formatting, metadata and reporting standards could facilitate seamless data integration and comparison across platforms and borders. Such a standardisation would help to create a more cohesive framework for toxicological data, making it easier for scientists and regulators worldwide to access and use information effectively.

Another important direction involves updating and expanding the databases to include emerging contaminants and advanced materials, such as nanoparticles and microplastics, which are of growing concern, but are currently underrepresented in toxicological datasets. Databases for advanced materials often do not contain comprehensive information on the exact parameters, such as the structure of a given substance, its purity, or nanoshape, which are necessary to assess toxicity and impact on organisms. Information on the toxicokinetic of new substances, such as the routes of absorption into the body, metabolism, distribution and excretion is also desirable, therefore, it is important that the results of experimental research, both in vivo and in vitro, provide comprehensive information about these substances, and this information could be placed in databases and described in detail. Expanding the databases to cover these materials would ensure that the regulatory community can keep up with technological advancements and emerging health threats.

Additionally, improving the accessibility and user interface of these databases is crucial. Simplifying the user experience through more intuitive design and better training resources can make these valuable tools more accessible to a broader range of users, including those who want to use knowledge of toxicology or information technology. Definitely, it will be helpful to use, among others, the tools presented in Table 2. This democratisation of data is vital to foster a broader engagement with toxicological information and enhance public health protection.

Lastly, fostering greater collaboration among international regulatory agencies, academic institutions, and industry stakeholders can improve the sharing and use of toxicological data. Through collaborative efforts, stakeholders can work towards a more integrated and comprehensive approach to managing chemical risks, which would be beneficial for aligning global safety standards and protecting public health across different regions.

These future directions aim to address the limitations of toxicological databases and envision a more dynamic, responsive, and collaborative approach to managing toxicological information in the digital age. By pursuing these goals, the toxicology community can improve the utility of these databases as indispensable tools in the ongoing effort to protect environmental and human health. 

**Table 1:** Types of resources used (suite of tools) with sources

Database	Information provided	Source
*Databases important for regulatory toxicology*		
ECHA	Provides information about classification, labeling, hazardous properties, safe use, SVHC identification, risk assessments and regulatory compliance	https://echa.europa.eu/pl/information-on-chemicals
ToxPlanet	Chemical hazards, toxicology, analytical methods, regulatory submissions, international scope, subscriptions, authoritative content, comprehensive data, support documents, diverse products, personalised search, usage management, instructional support, technical assistance.	https://www.enhesa.com/sustainablechemistry/our-solutions/toxplanet/
Spectral Database for Organic Compounds (SDBS)	Organic compound spectra, EI-MS, NMR, FT-IR, Raman, precision, reliability, free access.	https://www.library.ucsb.edu/spectral-database-organic-compounds-sdbs
CAMEO	Database enables the study and response to chemical emergencies, offering details on physical properties, health hazards, and emergency response recommendations.	https://cameochemicals.noaa.gov/
ISSTOX	Toxicological endpoints, mutagenicity, carcinogenicity, QSAR studies, Ames test results, in vivo assays, bioassay data, chemical structure searches, advanced data mining.	https://www.iss.it/isstox
Toxin and Toxin Target Database (T3DB)	Toxins, toxin targets, comprehensive data, chemical properties, toxicity metrics, molecular interactions, medical information, searchability, prediction, hazard awareness, diverse applications.	http://www.t3db.ca/
Tox21	Collaborative toxicity assessment, innovative test methods, robotics, high-throughput screening, predictive models, in vitro approaches, reduction of animal testing.	https://ntp.niehs.nih.gov/whatwestudy/tox21
International Uniform Chemical Information Database (IUCLID)	Chemical properties, hazard data, regulatory compliance, data submission, harmonized reporting, customizability, collaborative management.	https://iuclid6.echa.europa.eu/
CompTox chemicals dashboard	Supports research and regulatory efforts by providing comprehensive data on the chemistry, toxicity, and exposure of over a million chemicals, aiding in the identification of substances requiring further scrutiny and reducing reliance on animal testing.	https://www.epa.gov/comptox-tools/comptox-chemicals-dashboard
CompTox chemicals dashboard	Computational tools, chemistry, toxicity, and exposure information	https://www.epa.gov/comptox-tools/comptox-chemicals-dashboard
Acute toxicity database	The database contains results from 4901 aquatic toxicity tests by USGS CERC since 1965, used for evaluating chemical hazards in federal registration programs. It includes data on 410 chemicals and 66 species, established in 1986 for analyzing toxicity under various conditions.	https://www.cerc.usgs.gov/data/acute/acute.html
REACH	Chemical safety, regulation, toxicological studies, animal test data, alternative methods, read-across, computational tools, regulatory compliance.	https://echa.europa.eu/pl/information-on-chemicals/registered-substances
ChemIDplus	Research and regulatory compliance in environmental health and toxicology. It offers comprehensive details on chemical substances, including their properties, safety, toxicology, and regulatory statuses, facilitating the identification and comprehension of these substances.	https://pubchem.ncbi.nlm.nih.gov/source/ChemIDplus
Toxicity Reference Database (ToxRefDB)	Animal toxicity test results, curated data, in vivo studies, guideline compliance, predictive models, dose-response modeling, benchmark dose software, controlled vocabulary, standardized endpoints, inter-operability, quantitative utility.	https://www.epa.gov/chemical-research/downloadable-computational-toxicology-data
CEBS (Chemical Effects in Biological Systems database)	Enables the examination of toxicological impacts, such as carcinogenicity, acute toxicity, and genetic toxicity, by utilizing information from more than 11,000 exposure agents and 8,000 research studies.	https://cebs.niehs.nih.gov/cebs/
SciFinder	Chemical and bibliographic information, literature searches, patents, refined search capabilities, academic resource, online tutorials.	https://www.cas.org/cas-scifinder-discovery-platform/cas-scifindern
Australian National Industrial Chemicals Notification and Assessment Scheme (NICNAS)	Allows for the study and regulation of industrial chemical safety, detailing their properties, uses, and potential health and environmental impacts.	https://www.australianchamber.com.au/our-policies/work-health-safety/nicnas/
International Programme on Chemical Safety (IPCS)	Chemical risks, safety management, health effects, environmental impact, hazard characterization, and regulatory compliance.	https://www.ilo.org/resource/policy/international-programme-chemical-safety-ipcs
U.S. National Toxicology Program (NTP)	Environmental health effects, chemical exposure, laboratory experiments, epidemiological research, toxicity evaluation, NTP Technical Reports, functional omic technologies, transcriptomics, genomic dose-response modeling, BMDExpress 2.0 software, risk assessment.	https://ntp.niehs.nih.gov/
Comparative Toxicogenomics Database (CTD)	Enables the examination of chemical exposures and their impact on biology, aiding in the understanding of environmental health through the integration of information on chemicals, genes, traits, and illnesses.	https://ctdbase.org/
PubChem	Chemical structures, biological activities, safety information, GHS classifications, hazard statements, laboratory safety summaries, patents, literature citations, data integration, programmatic access.	https://pubchem.ncbi.nlm.nih.gov/
TOXBASE	Poison information, substance guidance, healthcare professionals, online accessibility, subscription options, clinical advice, dosing information, feedback mechanism, 24-hour helpline.	https://www.toxbase.org
Cosmetic Ingredient Review (CIR)	Information about cosmetic ingredients safety	https://www.cir-safety.org/
Cosmetic Ingredients (CosIng)	Information about cosmetic ingredients safety	https://ec.europa.eu/growth/tools-databases/cosing
International Fragrance Association (IFRA)	Information about fragrances ingredients	https://ifrafragrance.org/
International Fragrance Association (RIFM)	Information about fragrances ingredients	https://rifm.org/about-rifm
Scientific Committee on Consumer Safety (SCCS)	Scientific opinion about cosmetic ingredients in EU	https://health.ec.europa.eu/scientific-committees/scientific-committee-consumer-safety-sccs_en
Cosmetic Europe	Information about safety of cosmetics in EU	https://cosmeticseurope.eu/
Integrated in Silico Models for the Prediction of Human Repeated Dose Toxicity of COSMetics to Optimise Safety (COSMOS)	Comprehensive sources of information about cosmetic ingredients safety (including toxicological endpoints for safety assessment)	https://www.ng.cosmosdb.eu
EDETOX	Information about dermal absorption of chosen chemicals	https://research.ncl.ac.uk/edetox
DrugBank	Detailed drug data, including chemical, pharmacological, and pharmaceutical data among others	https://www.drugbank.com
European Medicines Agency (EMA) database	Information on human and veterinary medicines approved in the European Union	https://www.ema.europa.eu/en
FDA’s Adverse Event Reporting System (FAERS)	Database that contains information on adverse event and medication error reports submitted to FDA	https://www.fda.gov/drugs/questions-answers/fda-adverse-event-reporting-system-faers
FDAble	Searchable database of FDA approval documents and other FDA-related data	http://fdable.com
RxList.com	Provides detailed information about drug interactions, side effects, and uses	https://www.rxlist.com
Electronic medicines compendium (eMC)	Contains up to date, easily accessible information about medicines licensed for use in the UK	https://www.medicines.org.uk/emc
Databases important for forensic toxicology		
CFSRE	Reports on NPS, drug-impaired driving, drug-related deaths, drug-facilitated crimes, and advanced toxicology testing, detailing their impact on public safety and detection challenges.	https://www.cfsre.org
Erowid	Unbiased information on psychoactive substances and related topics.	https://erowid.org/)
DrugsData	Drug analysis results	https://www.drugsdata.org/index.php
NFLIS	Its primary objective is to provide accurate and timely information on drug trends, trafficking patterns, and the prevalence of various substances encountered in forensic investigations.	https://www.nflis.deadiversion.usdoj.gov/
EMCDDA	Provide the EU and its member states with accurate data on European drug issues and a unified framework for discussing drugs	https://www.emcdda.europa.eu/index_en
HighResNPS	Spectral database for NPS screening.	https://highresnps.com
ATSDR	Provides with reliable health information to prevent harmful exposures and diseases related to toxic substances.	https://www.atsdr.cdc.gov/
OSAC	Collection of chosen published and proposed standards for forensic science	https://www.nist.gov/organization-scientific-area-committees-forensic-science/osac-registry/
Databases important for food toxicology		
OpenFoodTox: chemical hazards database	Substance characterisation, EFSA findings, regulations toxicity, assessments reference points, exposure data	https://www.efsa.europa.eu/en/microstrategy/openfoodtox
Drugs and Lactation Database (LactMed^®^)	Medications, breast milk, adverse effects, nursing infants, alternative therapies, scientific literature	https://integrationacademy.ahrq.gov/resources/19391
FoodData Central (FDC)	USDA-based food composition information, five data categories, Foundation Foods (FF), Experimental Foods (EF), Standard Reference (SR) Legacy, Food and Nutrient Database for Dietary Studies (FNDDS), Global Branded Foods Products, Database (GBFPD)	https://fdc.nal.usda.gov
Evaluations of the Joint FAO/WHO Expert Committee on Food Additives (JECFA)	ECFA evaluations, flavours and food additive, contaminants, toxins, veterinary medications	https://www.fao.org/food-safety/scientific-advice/jecfa/en/
Inventory of Evaluations Conducted by the Joint Meeting on Pesticide Residues (JMPR)	Pesticide residues, assessments	https://apps.who.int/pesticide-residues-jmpr-database/Home/Range/All
Global Environment Monitoring System (GEMS)/Food Contamination Monitoring and Assessment Programme	Food contaminants, human exposure, public health	https://www.who.int/teams/nutrition-and-food-safety/databases/global-environment-monitoring-system-food-contamination
FDA toxicity databases	Toxicity databases, Hazardous Substances Data Bank (HSDB), Integrated Risk Information System (IRIS)	https://iuclid6.echa.europa.eu/pl/us-fda-toxicity-data
FADB (Food Additives Database)	FADB (Food Additives Database), EAFUS (All Added to Food in the United States) list, WHO and FAO food additive databases, toxicological assessments, chemical identifiers	https://food.ec.europa.eu/safety/food-improvement-agents/additives/database_en
Global Environment Monitoring System - Food Contamination Monitoring and Assessment Programme (GEMS / Food)	Dietary exposure evaluation, supranational diet models, chemical intake forecasting	https://www.who.int/teams/nutrition-and-food-safety/databases/global-environment-monitoring-system-food-contamination
US Food and Drug Administration’s (FDA) Toxic Elements in Food and Foodware and Radionuclides in Food Compliance Programme	Food safety oversight, chemical contaminants, toxic elements, radionuclides, dietary intake, public health protection	https://www.fda.gov/food/chemical-contaminants-pesticides/toxic-elements-foods-and-foodware
Pesticide Data Programme (PDP) of the US Department of Agriculture (USDA)	Pesticide residue monitoring, US food supply, dietary exposure evaluation, global promotion of US agricultural products, regulatory decision-making	https://www.ams.usda.gov/datasets/pdp
*Databases important for environmental toxicology and ecotoxicology*		
EPI Suite	Tool for estimating physicochemical properties, environmental fate, and ecotoxicity toxicity	https://www.epa.gov/tsca-screening-tools/epi-suitetm-estimation-program-interface
The National Institute of Standards and Technology (NIST)	Contains thermochemical, thermophysical, and ion energetics data	https://webbook.nist.gov/chemistry
Environmental Residue Effects Database (ERED)	Compiles data on the accumulation of harmful substances in tissues and their impact on various endpoints, such as mortality, growth, and physiological and biochemical responses	https://ered.el.erdc.dren.mil
The Environmental Protection Agency (EPA) established the ECOTOXicology database (ECOTOX)	Toxicity data sourced from the AQUIRE (aquatic life), PHYTOTOX (terrestrial plants), and TERRETOX (wildlife) databases	https://cfpub.epa.gov/ecotox/
EnviroTox	Contains aquatic toxicity effects records	https://envirotoxdatabase.org/
CalEcotox	Physiological and ecological parameters with toxicity information	https://ecotox.oehha.ca.gov/
ACToR (Aggregated Computational Toxicology Resource)	Data includes chemical structure, physico-chemical values, in vitro assay data and in vivo toxicology data	https://cfpub.epa.gov/si/si_public_record_report.cfm?dirEntryId=209598&Lab=NCCT
Distributed Structure-Searchable Toxicity (DSSTox)	Exceeds million substances which includes chemical lists of interest to EPA, other federal agencies, states, tribes, industry and other stakeholder groups	https://www.epa.gov/comptox-tools/distributed-structure-searchable-toxicity-dsstox-database

**Table 2:** Typical strengths and limitations of toxicological databases (qualitative overview)

Strengths of toxicological databases	
Support for regulatory decision-making	Toxicological databases are often used to the development and enforcement of chemical safety regulations. Can provide the critical data necessary for risk assessments and the evaluation of chemical hazards, thereby underpinning informed regulatory decisions.
Centralised repositories of toxicological information	These databases consolidate a wealth of information, including chemical properties, toxicological endpoints, and exposure data. This makes them invaluable resources for researchers, regulatory agencies, and industry stakeholders seeking to understand the potential impacts of chemical substances on health and the environment.
Support for non-animal testing approaches	These databases can provide access to extensive datasets to advance computational models and in vitro tests. This aligns with the ethical push to reduce animal testing in toxicological research and to promote more humane scientific practices.
Facilitation of international collaboration	The global scope of some toxicological databases facilitates the exchange of data between borders. This international collaboration helps to harmonise safety standards and regulatory practices, enhancing global public health protection.
*Limitations of toxicological databases*	
Accessibility and user interface issues	Many databases suffer from complex or nonintuitive user interfaces that can hinder efficient access and use, especially by those without specialised training. This limits the usability of the databases and can slow research and regulatory processes.
Inconsistencies and standardisation issues:	The lack of standardised formats across different databases leads to inconsistencies that complicate data integration and comparison. These inconsistencies can result in inefficiencies and an increased potential for errors in toxicological assessments.
Data gaps and quality issues	Some toxicological databases may have significant data gaps, particularly with regard to newer or less studied chemicals. Furthermore, data quality can vary, and some databases rely on outdated or poorly validated information.
Maintenance and update challenges	Keeping databases up-to-date with the latest scientific findings and regulatory changes requires substantial ongoing effort and resources. Inadequate maintenance can make the data less relevant and reduce the effectiveness of the databases as regulatory tools.

## Supplementary Information

Below is the link to the electronic supplementary material.

Supplementary Material 1

## Data Availability

The data, analytic methods, and study materials that support the findings of this study are available from Kamil Jurowski (toksykologia@ur.edu.pl) on reasonable request.

## Abbreviations

ADI: Acceptable daily intake
AI: Artificial intelligence
BER: Bioactivity/exposure ratio
BMD: Benchmark dose
CLP: Classification, labelling and packaging
CMR: Carcinogenic, mutagenic, or reproductive toxicants
DBMS: Database management system
DBS: Database system
ECHA: European chemicals agency
EEA: European economic area
EFSA: European food safety authority
EMA: European medicines agency
EU: European union
FDA: Food and drug administration
IATA: Integrated approaches to testing and assessment
iTTC: Internal TTC
LD50: median lethal dose, rate 50%
ML: Machine learning
NGRA: Next generation risk assessment
NOAEL: No observed adverse effect level
NPS: New psychoactive substances
OECD: Organisation for economic cooperation and development
NAM: New approach methods
PBT: Persistent, bioaccumulative, and toxic
PBTK: Physiologically based toxico kinetics
PNEC: Predicted no effect concentration
QSAR: Quantitative structure-activity relationship
REACH: Registration, evaluation, authorisation, and restriction of chemicals
SVHCs: Substances of very high concern
TDI: Tolerable daily intake
TTC: Threshold of toxicological concern
vPvB: Very persistent and very bioaccumulative
