# Supplementary material for: Navigating through toxicological data: review of toxicological databases and sources in the interdisciplinary context including—chemicals, cosmetics, pharmaceuticals and forensics
Source: Arch Toxicol. 2026 Apr 20;100(7):2779–812. doi: 10.1007/s00204-026-04379-y (PMC13309490; doi:10.1007/s00204-026-04379-y)
Supplement: Supplementary file 1 — Supplementary Material 1 [file 204_2026_4379_MOESM1_ESM.docx]

29^th^, January 2026

Hradec Kralove

Czech Republic

To: **Editor of Archives of Toxicology**

*Resubmission of Revised Manuscript (New Submission)*

Dear Editor-in-Chief,

We would like to submit a revised version of our manuscript to Archives of Toxicology as a **new submission**, following the editorial decision on our previous manuscript (Manuscript No. **ATOX-D-25-01302**).

With reference to the previous submission No. **ATOX-D-25-01302** and the information that the manuscript was rejected but we were encouraged to resubmit the manuscript, we are submitting a revised version that takes into account all reviewer comments, along with detailed responses to these comments.

We carefully and thoroughly addressed all concerns raised by the reviewers. In particular, the manuscript was substantially shortened, redundant content removed, figures eliminated, tables reorganized, and several sections rewritten to improve clarity, structure, and scientific rigor in line with the high standards of Archives of Toxicology. We believe that the revised manuscript now represents a concise, coherent, and substantially improved review.

For transparency, we provide below the reviewers’ comments and our corresponding responses, exactly as prepared during the revision process:

**Reviewer 1:**

This review summarizes databases for a wide range of areas in toxicology. Overall, the review is well written, but there are many repetitions in terms of content (e.g. p 7 line 8-12, doubling with sentence before). In a revision, I would also recommend shortening the text. In the beginning of the Review, there is a constant repetition of information. In my opinion, the illustrations are not really necessary for understanding the text, and I also find the choice of illustrations (some of which are screenshots) unsuitable for a journal. Overall, I can say that the review is a very comprehensive compilation and overview, but in my opinion, it does not meet the high scientific standards that Archives of Tox expects of its reviews.

**Author’s response:** We are very grateful for your comments, which helped improve our manuscript. We have shortened the text to eliminate duplicate information and removed figures to ensure the text meets the journal's requirements.

**Reviewer 2:**

This is a very comprehensive manuscript that summarized the toxicological databases for Chemicals, Cosmetics, Pharmaceuticals, and Forensics. Concise summarization of these databases and their utilization would be useful for Toxicologists, Pharmacologists, and Regulatory Agents; however, this review is overwhelming and redundant. The significant condensation and reorganization are needed to improve the presentation. Listed below are specific comments:

1. This article from Introduction to Conclusion had 21600+ words, plus three big Tables and 7 Figures, much longer than any review-type articles.

**Author’s response:** Thank you for your comment, we have shortened the text and removed the figures and one table.

2. Are Figure 1-7 necessary for this review? These "screen-cut" Figures may add more burdens to this already tedious text, raising more questions than answers.

**Author’s response:** Thank you for your comment, we have removed the figures.

3. Table 2 provided useful information on Toxicology databases. However, it could be grouped by the database importance, by alphabetical order, or more appropriately by the orders of the text subtitles, i.e., Regulation (35: Chemical-21, Cosmetics-8, Drugs-6), Toxicology (7), Food (11) and Environment (9).

**Author’s response:** Thank you for your comment, we have reworked the table as per the reviewer's suggestion.

4. Table 1 summarized databases and descriptions contained in ToxPlanet. Some of them are already described in other subtitles, such as AcuteTox, ATSDR, CTD, Pubchem, T3DB, and NTP, NTP was even described twice in Table 1, and duplicated in Table 2. Such redundant presentation should be avoided.

**Author’s response:** Thank you for this comment, as Table 1 duplicated information already contained in the text, we decided to remove it to avoid such redundancy.

5. Table 3 compared the "Strengths" and "Weaknesses" of toxicological databases. However, the criteria are quite different across these databases. For example, the regulation criteria of TCDD in Europe is stricter than that in USA. Such evaluation appears to be too general to be specific.

**Author’s response:** Thank you for your comment. This table was intended to provide a concise overview of typical database advantages and limitations, not to compare them with each other. Therefore, we have slightly modified it and added appropriate statements in the text.

6. Others, for example: a. Abstract, Line 7 and 16: "in-formation" should be "information". There are many such word separations in the paper, which should be avoided.

**Author’s response:** Thank you for this comment, these pauses were caused by the text editing program that split words at the ends of lines, we have corrected it.

b. Introduction, Line 10: "Yang et al., n.d." is invalid. Please make sure the reference citation is correct.

**Author’s response:** Thank you for your comment, we have corrected the citation.

c. Section 3.2.5 SCCS, Page 23, line 6-53, the text presentation style is quite different that other sections.

**Author’s response:** Thank you for your comment, we have rewritten this paragraph.

d. Table 2, why the "Comptox Chemicals Dashboard", "OpenFoodTox-Chemical Hazards Database", and "Drugs and Lactation Database (LactMed)" not in BOLD as others?

**Author’s response:** Thank you for this comment, as per your previous comment we have reworked the table, also taking into account the new text formatting.

We sincerely appreciate the constructive feedback from the reviewers and the encouragement to resubmit. We believe that the revised manuscript now meets the scientific and editorial expectations of *Archives of Toxicology* and hope it will be suitable for reconsideration.

Thank you very much for your time and consideration.

Sincerely,

**Kamil Kuca**

(on behalf of all authors)

**prof. Kamil Kuca**

t: +420 603 289 166

e-mail: [kamil.kuca@uhk.cz](mailto:kamil.kuca@uhk.cz); [kamil.kuca@fnhk.cz](mailto:kamil.kuca@fnhk.cz)

social networks:  [LinkedIn](https://www.linkedin.com/profile/view?id=AAEAAAPjZrgBf8duqjHl_uLZ1mS7n_WQ-nI_c6Q) / [ResearchGate](http://www.researchgate.net/profile/Kamil_Kuca)
